# Phytocompounds with immunomodulatory activities: mechanisms, therapeutic potential, and clinical prospects

**DOI:** 10.1007/s10787-026-02305-3

**Published:** 2026-07-13

**Authors:** Noha A. Mehana, Nourhan Ibrahim Morad, Rehab Mekky, Amal M. Mahfoz, Riham A. El-Shiekh, Mahmoud A. Elrebehy

**Affiliations:** 1 Biochemistry Department, Faculty of Pharmacy, Cairo University, Cairo, Egypt; 2 Department of Pharmacognosy and Natural Products, Faculty of Pharmacy, Menofia University, Shibin Elkom, Menofia, 32511 Egypt; 3 Biochemistry Department, Faculty of Pharmacy, Badr University, Assiut, Egypt; 4 Saudi Toxicology Society, Department of Pharmacology and Toxicology, College of Medicine, Umm Al Qura University, Makkah, Saudi Arabia; 5 Egyptian Drug Authority, Cairo, Egypt; 6 Department of Pharmacognosy, Faculty of Pharmacy, Cairo University, Kasr El-Aini Street, Cairo, 11562 Egypt; 7 Department of Biochemistry, Faculty of Pharmacy, Galala University, New Galala, Suez, 43713 Egypt

**Keywords:** Immune system, Immunomodulatory agents, Immune disorders, Phytocompounds, Natural bioactive compounds, Innate and adaptive immunity

## Abstract

The immune system is essential for host defense, immune surveillance, and maintenance of physiological homeostasis; its dysregulation underlies a broad spectrum of disorders, including autoimmune, inflammatory, infectious, neoplastic, and immunodeficiency diseases. Despite advances in modern immunotherapy, current treatments remain limited by suboptimal efficacy, adverse effects, resistance, and high cost, necessitating the development of safer and more effective immunomodulatory strategies. This review provides a comprehensive and integrative synthesis of phytocompounds as emerging immunomodulators, with emphasis on their mechanisms and translational potential. A structured literature search was conducted using major databases (PubMed, Scopus, and Web of Science) covering studies published between 2000 and 2025. We systematically analyze major classes of bioactive phytocompounds, including polysaccharides, flavonoids, polyphenols, terpenoids, and alkaloids, highlighting their capacity to modulate both innate and adaptive immune responses. These compounds exert pleiotropic effects through regulation of key immune cells; macrophages, dendritic cells, T and B lymphocytes, and natural killer cells, and modulation of critical signaling pathways such as NF-κB, MAPKs, JAK/STAT, and Nrf2. Importantly, this review bridges preclinical findings with clinical evidence, critically evaluating their therapeutic potential in autoimmune diseases, chronic inflammatory conditions, infections, and immune-mediated metabolic disorders. Despite promising outcomes, challenges related to safety and clinical validation remain. Future research should focus on pharmacokinetics, standardized dosing, and large-scale clinical trials to support their therapeutic translation.

**Graphical abstract:**

**Figure:**
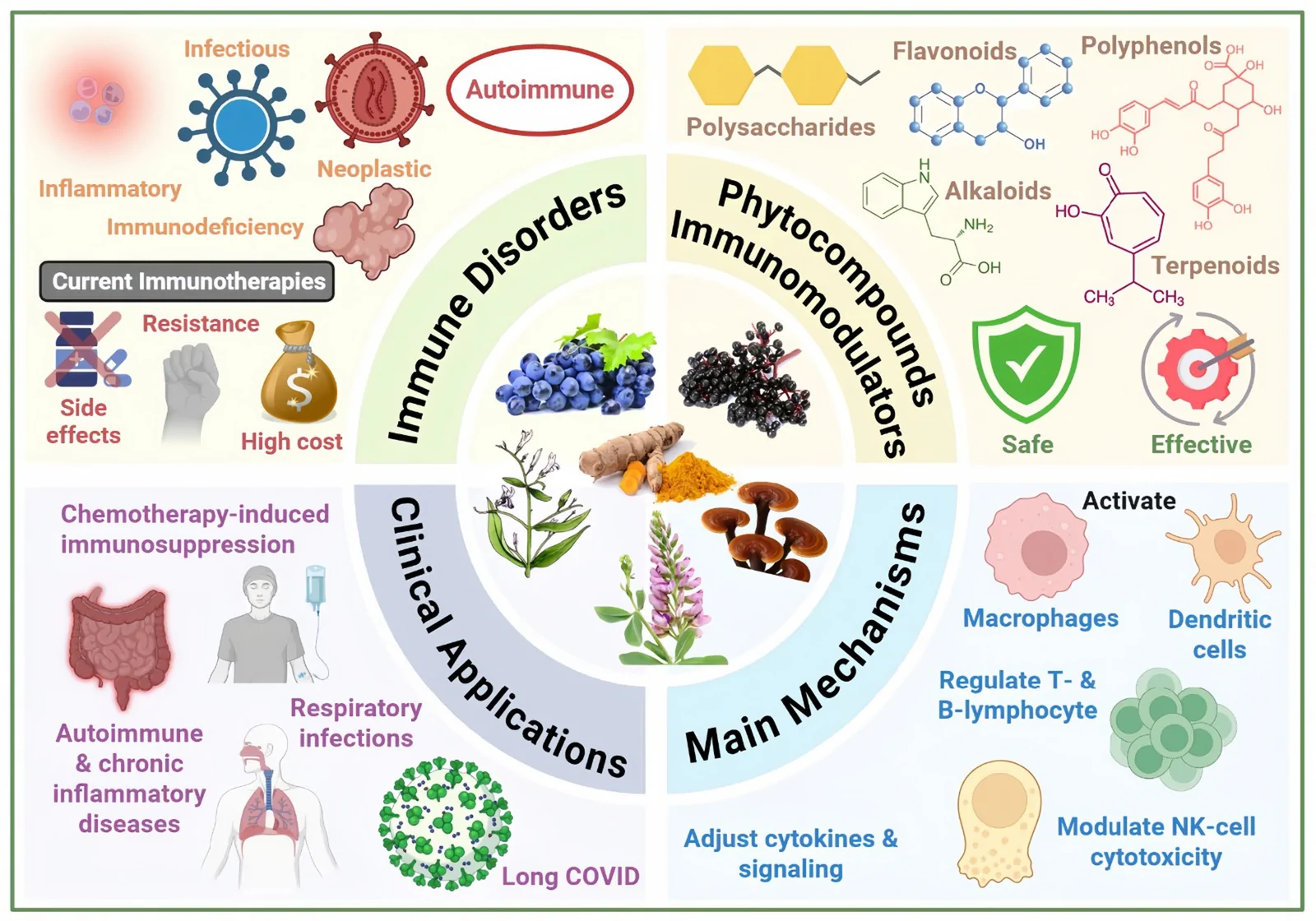

## Introduction

The immune system is a highly coordinated network of innate and adaptive components that maintains immune tolerance and protects against infections, environmental stressors, and malignancy (Wang et al. 2024a). Innate immunity represents the first line of defense through physical barriers and non-specific cellular responses, including granulocytes, macrophages, and natural killer (NK) cells, whereas adaptive immunity provides antigen-specific responses mediated by T and B lymphocytes (Domínguez-Andrés et al. 2023; Ochando et al. 2023).

Dysregulation of immune responses contributes to a wide spectrum of pathological conditions, including autoimmune diseases, immunodeficiency, hypersensitivity, autoinflammatory disorders, and malignancies (Kumar et al. 2025). The global burden of immune-related disorders is increasing, driven by environmental pollution, genetic susceptibility, lifestyle changes, microbiome alterations, and population aging (Caliskan, Brown, and Maranville 2021). Notably, autoimmune diseases affect approximately 10% of the population worldwide and represent a leading cause of chronic morbidity, particularly among women (Goulmamine et al. 2024; Conrad et al. 2023).

Immunomodulatory agents, classified as immunosuppressants or immunostimulants, play a central role in restoring immune balance (Gurjar and Pal 2021). While immunosuppressive therapies are essential in transplantation and autoimmune diseases, immunostimulants enhance host defense mechanisms in immunocompromised individuals (Szumilas et al. 2023; Sharma et al. 2024). Despite advances in immunotherapy, including corticosteroids, biologics, and small-molecule inhibitors, current treatments remain limited by adverse effects, incomplete efficacy, resistance, and high cost (Jung and Kim 2022; Bhagavati 2021). These limitations highlight the need for safer, more targeted, and homeostasis-oriented therapeutic approaches (Pillai and Antony 2024).

In this context, plant-derived bioactive compounds have gained increasing attention as potential immunomodulators. Phytocompounds, including polysaccharides, flavonoids, polyphenols, terpenoids, alkaloids, and glycosides, exhibit multifaceted effects on the immune system. These compounds enhance host defense while suppressing aberrant immune activation (Di Sotto et al. 2020). Their pleiotropic actions involve regulation of macrophages and dendritic cells, modulation of NK cell activity, and control of T- and B-lymphocyte responses, in addition to influencing key signaling pathways such as NF-κB, MAPKs, JAK/STAT, and Nrf2 (George et al. 2020; Zehra et al. 2025). Furthermore, emerging evidence highlights their role in modulating inflammatory pathways and the gut microbiome (Hooda et al. 2024).

Recent studies support the therapeutic potential of phytocompounds in immune-related conditions, including immunodeficiency, autoimmune diseases, chronic inflammation, and viral infections (Nisar et al. 2023; Pillai and Antony 2024). For instance, *Astragalus membranaceus*, *Panax ginseng*, and *Ganoderma lucidum* enhance immune competence in immunosuppressed states, whereas *Andrographis paniculata* and *Sambucus nigra* improve antiviral responses (Tang et al. 2024; Wang et al. 2025; Harnett et al. 2020; Kamal Perera, Dilanthi Diddeniya, and Banda Meedeniya 2022). In addition, compounds such as curcumin, resveratrol, and epigallocatechin gallate (EGCG) exert immunoregulatory and anti-inflammatory effects in autoimmune disorders (Mangla et al. 2025; Peng et al. 2021; Wątroba and Szukiewicz 2025).

This review provides an integrative and updated synthesis of phytocompounds as immunomodulatory agents, emphasizing their cellular and molecular mechanisms, clinical relevance, and translational potential. By bridging preclinical evidence with clinical insights, this work aims to highlight the therapeutic promise of phytochemicals and identify key challenges and future directions for their development as complementary or alternative immunotherapeutic strategies.

## Methodology

This review was conducted using a structured and comprehensive literature search strategy to identify relevant studies on phytocompounds with immunomodulatory activities. Electronic databases, including PubMed, Scopus, Web of Science, and Google Scholar, were narratively searched for articles published between 2000 and 2025, with particular emphasis on recent studies from 2020 to 2025 to ensure up-to-date scientific and clinical relevance. The search was performed using combinations of keywords and Boolean operators, including: *“phytocompounds*,*” “natural products*,*” “immunomodulation*,*” “immune disorders*,*” “cytokines*,*” “NF-κB*,*” “JAK/STAT*,*” “MAPK signaling*,*” “autoimmune diseases*,*” and “inflammation.”* Reference lists of relevant articles were also manually screened to identify additional studies.

Eligible studies included in vitro experiments, in vivo animal studies, and clinical trials that investigated the immunomodulatory effects, molecular mechanisms, therapeutic potential, or safety of plant-derived bioactive compounds. Review articles were used selectively for background information and contextual support. Studies were excluded if they were non-English, duplicates, lacked sufficient methodological detail, or were not directly related to immunomodulation. Priority was given to studies with robust experimental design, clear mechanistic insights, and clinically relevant outcomes.

The collected data were analyzed and integrated qualitatively, with a focus on mechanistic pathways, therapeutic applications, and translational relevance, aiming to provide an integrative and critical overview of current evidence.

## Immune-related disorders: classification, burden, and determinants

### Pathophysiology and classification of immune disorders

The immune system depends on tightly regulated cellular and molecular responses to maintain host defense and immune homeostasis (Wang et al. 2024b). Dysregulation of this system may manifest as immunodeficiency, autoimmunity, autoinflammatory disorders, hypersensitivity diseases, or neoplastic immunity diseases (Starshinova et al. 2025).

Immunodeficiency disorders may be primary, resulting from inherited genetic defects, or secondary, arising from acquired causes such as infections; both forms impair pathogen clearance and increase susceptibility to recurrent or opportunistic infections (Ahmed et al. 2022; Wang et al. 2025).

Autoimmune diseases arise when immunological tolerance is disrupted, leading to immune responses against self-antigens (Sasikala and Reema Kumari 2023). Depending on the extent of tissue involvement, they could be organ-specific or systemic diseases. Their pathogenesis commonly involves autoreactive T cells, autoantibodies, dysregulated cytokine production, and activation of inflammatory cascades (Pisetsky 2023; Xiang et al. 2023).

Autoinflammatory disorders are primarily driven by dysregulated innate immune responses and often present clinically with recurrent inflammatory episodes and elevated acute-phase responses. Key mechanisms include aberrant inflammasome activation, excessive cytokine production, and dysregulated Toll-like receptor (TLR) signaling (Starshinova et al. 2025).

Collectively, the heterogeneity of these disorders highlights the critical need for various therapeutic strategies to restore immune homeostasis.

### Global epidemiology and burden of immune-related disorders

The epidemiology of immune-related disorders has changed substantially in recent years, with notable shifts in disease burden, population distribution, and clinical recognition (Nautiyal et al. 2024). Despite their complex pathogenesis, these disorders are common and affect almost 10% of the world’s population (Conrad et al. 2023). Many of these disorders disproportionately affect women, who account for approximately 80% of cases (Goulmamine et al. 2024).

For example, rheumatoid arthritis affected an estimated 17.6 million people globally in 2020 and is projected to increase substantially by 2050 (Song et al. 2025). Their burden also varies across regions and populations; for instance, the reported birth prevalence of severe combined immunodeficiency (SCID) has been estimated at approximately 1/100,000 in some parts of the United States, compared with 20/100,000 in the Middle East (Aranda et al. 2021).

Beyond their impact on morbidity and mortality, immune-related disorders impose substantial socioeconomic burdens through increased healthcare costs, long-term treatment requirements, reduced productivity, and impaired quality of life. These consequences are particularly evident in chronic autoimmune and inflammatory diseases that require lifelong monitoring and management (Miller 2023).

These epidemiological trends highlight the growing clinical burden of immune disorders and reinforce the need for safer and more effective therapeutic strategies.

### Risk factors and mechanistic drivers of immune dysregulation

A wide range of factors contribute to immune dysregulation, including genetic susceptibility, environmental exposures, and lifestyle-related influences. Environmental contributors such as air pollution, allergen exposure, certain medications, infections, and altered microbial interactions, including Epstein–Barr virus (EBV), can promote immune dysregulation by disrupting epithelial barriers, enhancing oxidative stress, and triggering persistent inflammatory or autoreactive responses (Houen and Trier 2021).

Geographic and sunlight-related factors may further shape these effects by influencing microbial exposure and vitamin D status. In addition, limited microbial exposure, as proposed by the hygiene hypothesis, may impair immune education and increase susceptibility to autoreactivity and immune imbalance (Zorena et al. 2022; Cui et al. 2025; Kumar et al. 2025).

Lifestyle-related factors such as obesity, cigarette smoking, psychological stress, vitamin D deficiency, tumors, and alterations in the human microbiome have also been implicated in immune dysregulation through mechanisms including chronic low-grade inflammation, cytokine imbalance, oxidative stress, and impaired regulatory immune responses. Vitamin D contributes to immune homeostasis through multiple regulatory mechanisms, including modulation of T-cell responses and inflammatory mediators (Bhagavati 2021; Pisetsky 2023). Diet composition may further influence immune homeostasis through modulation of the gut microbiota, which regulates metabolites such as tryptophan derivatives and short-chain fatty acids (SCFAs) that are essential for immune cell function (Brown et al. 2020).

Sex-based differences also influence immune regulation, with females generally exhibiting more robust immune responses than males, likely due to differences in sex hormones and sex chromosome complement. Endocrine transitions such as menopause may further increase susceptibility to autoimmune disorders (Motta et al. 2025).

**Fig. 1:**
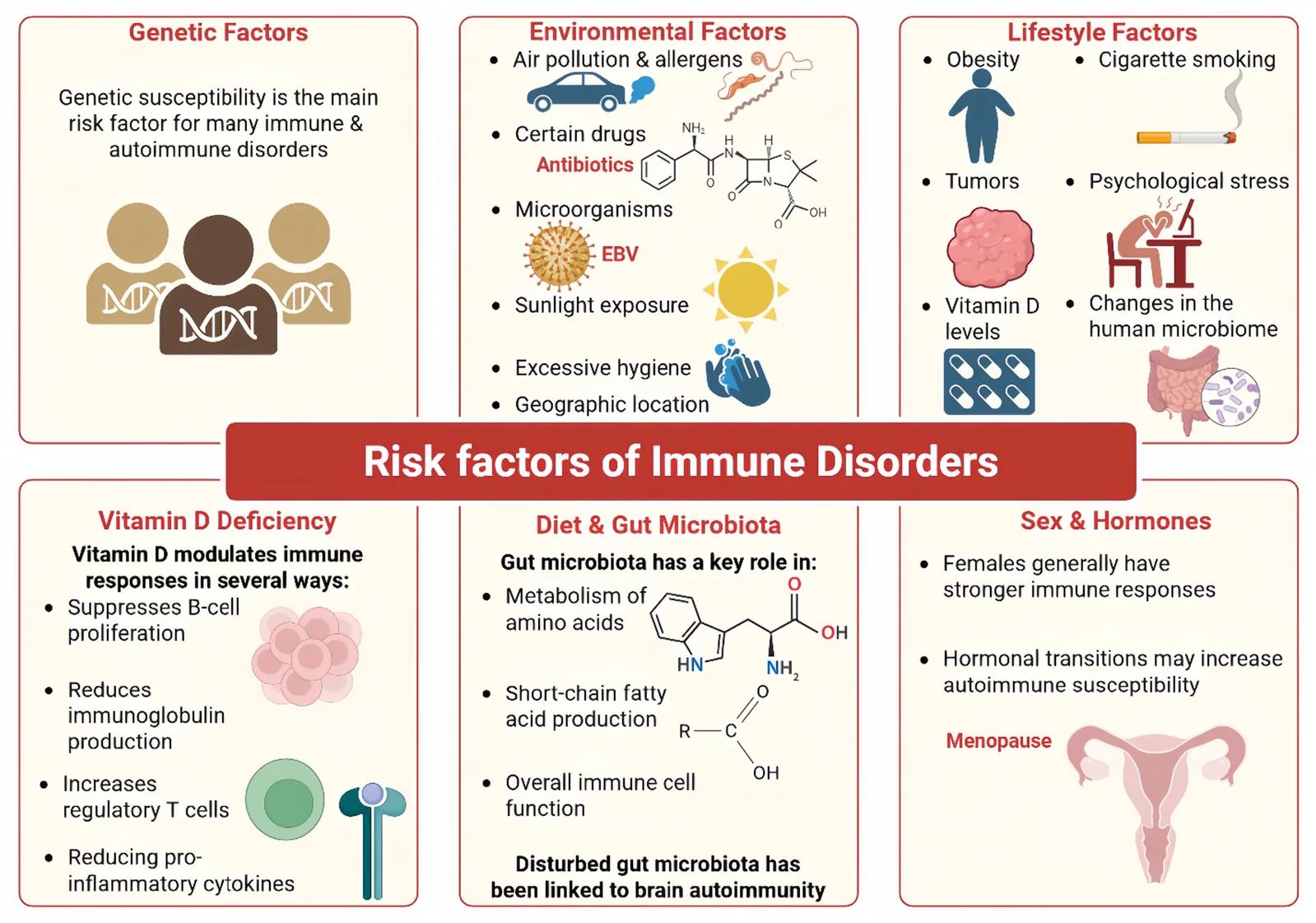
Major genetic, environmental, lifestyle, hormonal, microbiota-related, and vitamin D–associated factors involved in the development and progression of immune and autoimmune disorders. Created with BioRender.com

Collectively, these interacting genetic, environmental, and lifestyle-related factors contribute to immune dysregulation and influence both disease susceptibility and progression (Fig. 1).

### Current therapeutic strategies and limitations

Early therapeutic intervention is important for limiting organ damage and reducing the risk of persistent or lifelong disease activity. Current therapeutic strategies for autoimmune disorders rely mainly on broad immunosuppression, with plasma exchange used in selected settings to remove pathogenic autoantibodies; however, such approaches are often limited by non-specific immune suppression and treatment-related toxicity. Conventional immunosuppressants such as mycophenolic acid (MPA) and mizoribine remain useful in organ transplantation and autoimmune disorders, although their clinical benefit may be constrained by systemic adverse effects and long-term immune suppression (Ali and Ben-Sahra 2023).

Disease-specific approaches have also expanded; for example, immune thrombocytopenia (ITP) is commonly managed with corticosteroids, intravenous immunoglobulin (IVIG), and thrombopoietin receptor agonists (TPO-RAs), some of which exert limited immunosuppressive effects (Madkhali 2024). Tregitopes peptides are novel agents that emerged for restoring immune balance in clinical applications (Javidan et al. 2024). In parallel, polysaccharide analogues have been recognized as promising therapeutic agents due to their capacity to modulate the immune response through cellular, molecular, and genetic mechanisms (Huang et al. 2025b).

In autoimmune disorders of the central and peripheral nervous systems, therapeutic options include sphingosine 1-phosphate receptor (S1PR) modulators such as fingolimod, proteasome inhibitors, and agents that suppress T- or B-lymphocyte proliferation (Bhagavati 2021). In contrast to conventional immunotherapies, bioactive phytocompounds from medicinal plants have attracted increasing interest because many exert multi-target immunomodulatory effects, including regulation of inflammatory mediators, modulation of innate immune signaling, and concurrent antioxidant activities (Sekkout et al. 2024). Recent reviews on naringenin and eugenol further support this concept, highlighting their ability to modulate multiple pathways linked to inflammation, apoptosis, angiogenesis, and tumor progression, while also emphasizing persistent limitations such as poor bioavailability, formulation challenges, and insufficient clinical validation (Noman et al. 2025a; Noman et al. 2025b).

Hematopoietic stem cell transplantation (HSCT) has been implicated as a therapeutic option for many immunodeficiency disorders; however, it has caused adverse effects such as organ toxicity and graft failure. Gene therapy is also emerging as a promising strategy for otherwise incurable immunodeficiency disorders (Eshghi et al. 2025). Nevertheless, translating mechanistic insights into durable, clinically effective immunomodulatory therapies remains challenging due to disease heterogeneity, variable patient responses, and safety concerns (Jain et al. 2024).

Despite the availability of various therapeutic approaches and the advances in immunopathology, current treatment options primarily manage symptoms rather than addressing the underlying cause. In addition, many of them are associated with significant side effects that negatively affect quality of life (QOL) and daily activities. Increasing emphasis is therefore being placed on targeted and precision-based immunotherapeutic strategies that can restore immune homeostasis while minimizing systemic toxicity.

Given these limitations, increasing attention has been directed toward natural immunomodulators, particularly phytocompounds, as potential adjuncts or alternatives that may offer broader mechanistic coverage with potentially lower systemic toxicity, although stronger clinical evidence is still needed.

## Cellular and molecular basis of phytocompound-mediated immunomodulation

Cellular and molecular mechanisms of natural immunomodulators encompass coordinated interplay among innate and adaptive immune cells, soluble mediators, and hematopoiesis, leading more often to context-dependent “immune tuning” rather than simple, nonspecific stimulation (Borchers et al. 2008; Goodridge et al. 2009; Ríos 2010; Brindha 2016; Behl et al. 2021; Kim et al. 2022; Zheng et al. 2023; Guo et al. 2026).

### Macrophage and phagocyte activation

Macrophages, along with other professional phagocytes such as neutrophils, monocytes, and dendritic cells (DCs), are central to innate immunity. Many phytocompounds target these cells to enhance pathogen clearance, antigen presentation, and the early cytokine response. These compounds exert their effects by modulating multiple signaling networks involved in innate immune activation and inflammatory regulation. (Borchers et al. 2008; Jantan et al. 2015; Netea et al. 2016; Kim et al. 2022; Strzelec et al. 2023; Rivero-Pino and Montserrat-de la Paz, 2024).

For instance, Astragalus polysaccharides (APS) enhance innate immune responses by stimulating macrophage phagocytosis, upregulating pattern recognition receptors (PRRs) such as TLR4 and Dectin-1, and increasing the production of pro-inflammatory cytokines like TNF-α and IL-1β in both mouse and human macrophages (Li and Wu 2021). APS also promotes dendritic cell (DC) maturation, increasing MHC II expression and co-stimulatory molecules such as CD80/CD86, which enhances antigen presentation. Furthermore, APS support T cell proliferation, leading to increased CD4^+^ and CD8^+^ T cell numbers, as well as augmented antigen-specific IgG production, thereby reflecting a coordinated enhancement of both cellular and humoral immunity (Cho and Leung 2007; Di Sotto et al. 2020; Alanazi et al. 2023).

Furthermore, β-glucans and triterpenes from Ganoderma, Grifola frondosa, and Lentinula edodes activate macrophages and dendritic cells (DCs) through Dectin-1, complement receptor 3 (CR3), and TLRs. This activation enhances phagocytosis, antigen presentation, and cytokine release. The Syk–CARD9 axis, along with downstream signaling through NF-κB and MAPKs, promotes ROS and NO generation, which fosters a pro-inflammatory microenvironment bridging innate and adaptive immunity (Brown and Gordon 2005; Borchers et al. 2008; Goodridge et al. 2009). This complex cascade not only enhances pathogen clearance but also coordinates immune responses that drive adaptive immune activation, crucial for effective immune defense (Brown and Gordon 2005; Netea et al. 2016; Zehra et al. 2025).

Similarly, Andrographolide, a key phytocompound from Andrographis paniculata, exerts dual anti-inflammatory and immunomodulatory actions on macrophages. It attenuates excessive NF-κB-mediated production of pro-inflammatory cytokines such as TNF-α and IL-6, while preserving or enhancing phagocytic activity and promoting IL-12 production. These actions support more efficient yet controlled innate immune responses to both respiratory and systemic pathogens, preventing excessive inflammation and fostering immune tolerance (Islam et al. 2018; Zeng et al. 2022). This balanced immune response is critical in autoimmune diseases and infection control (Aromdee et al. 2011; Islam et al. 2018; Andrés et al. 2022).

Consistently, Propolis flavonoids and phenolic acids modulate macrophage and neutrophil functions by increasing phagocytic capacity, enhancing bactericidal activity, and fine-tuning pro-inflammatory cytokines secretion via NF-κB, Nrf2, and p38 MAPK pathways (Sforcin and Bankova 2011; Sforcin 2016; Xu et al. 2022b). These macrophage-directed actions underpin the observed benefits of propolis in both local and systemic infections, particularly within the oral and respiratory mucosa (Otręba et al. 2022; Ożarowski and Karpiński 2023).

**Fig. 2:**
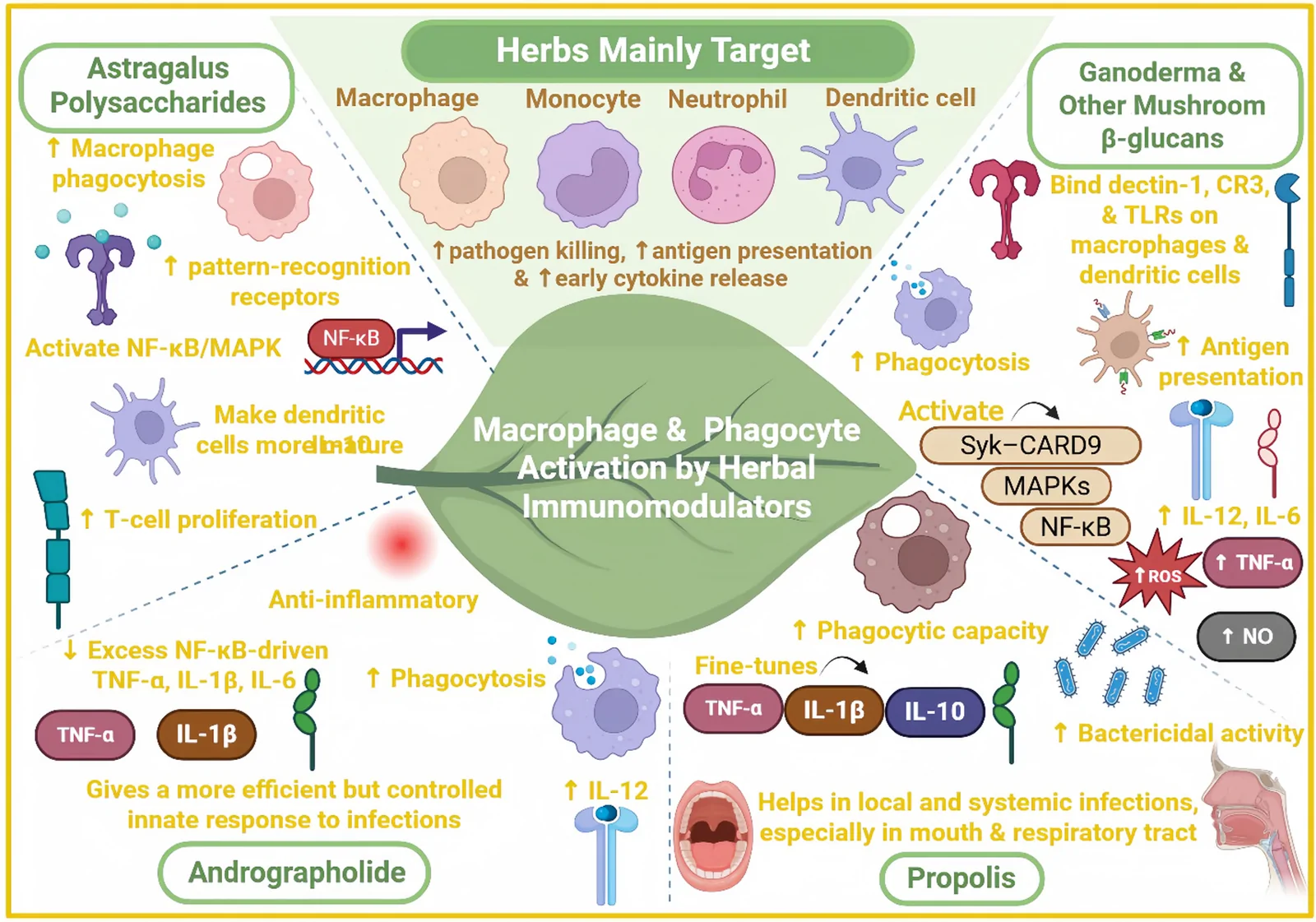
Mechanisms of macrophage and phagocyte activation by selected herbal immunomodulators. Representative phytocompounds enhance innate immune responses by promoting phagocytosis, antigen presentation, cytokine production, and antimicrobial activity through modulation of key immune signaling pathways. Created using BioRender.com

Taken together, these herbs increase recruitment, pathogen uptake, and early cytokine production by phagocytes while, in some cases, dampening pathological inflammation through antioxidant and anti-inflammatory signaling (Borchers et al. 2008; Sforcin and Bankova 2011; Sforcin 2016; Rivero-Pino and Montserrat-de la Paz, 2024) (Fig. 2).

### NK cell activation and nonspecific defense

NK cells play a critical role in the body’s early defense against viruses and tumors. Several phytocompounds enhance NK-cell-mediated immunity through regulation of signaling pathways involved in proliferation, cytotoxicity, and cytokine production (Borchers et al. 2008; Hyun et al. 2021; Wu et al. 2025).

Specifically, β-glucan-rich preparations from Ganoderma and other medicinal mushrooms enhance NK cell activity against tumor and virus-infected targets in both in vivo and in vitro models. These effects are mediated by increased cytokine production, such as IFN-γ and TNF-α, which trigger NK cell proliferation and cytotoxicity (Matsui et al. 2001; Orange and Ballas 2006; Borchers et al. 2008; Malak et al. 2025). Furthermore, β-glucans activate dectin-1 and TLR signaling, which contribute to enhanced NK function through MAPK and NF-κB pathways. This complex cascade not only enhances pathogen clearance but also coordinates immune responses that drive adaptive immune activation, crucial for effective immune defense (Turnbull 2023; Shankar et al. 2024).

Another example is Ginsenosides which enhance NK cell cytotoxicity and modulate NK subset distribution, with in vitro and in vivo data showing increased expression of activation markers and IFN-γ production, partly via PI3K/AKT and JAK/STAT signaling (Hyun et al. 2021). Clinical and preclinical studies indicate that ginseng supplementation increases NK cell numbers and activity in peripheral blood, contributing to improved antiviral and antitumor responses (Kim et al. 2022; Paudel et al. 2023; Lee et al. 2025).

Recent evidence indicates that monoterpenes such as thymol and carvacrol modulate key immune signaling pathways including NF-κB, MAPK/ERK, PI3K/AKT, and JAK/STAT, leading to altered cytokine profiles and enhanced immune regulation; studies also highlight their potential synergy with conventional therapies, though challenges such as bioavailability and stability limit clinical translation (Noman et al. 2025c).

Additionally, Elderberry anthocyanins and flavonoids show antiviral effects by inhibiting viral attachment and replication and promoting pro-inflammatory cytokines production by monocytes and other innate cells at early infection stages (Roschek et al. 2009; Tiralongo et al. 2016; Hawkins et al. 2019). Although direct human data on NK cells are limited, elderberry extracts may indirectly enhance NK activation and recruitment through these innate cytokine changes and improved antioxidant defenses (Hawkins et al. 2019).

By augmenting NK cytotoxicity and the cytokines that govern NK function, these natural agents reinforce nonspecific resistance to infections and malignancies, particularly at early disease stages or under immunosuppressive stress (Hyun et al. 2021).

### T- and B-cell modulation and antibody responses

Several phytocompounds modulate adaptive immunity by influencing T- and B-cell activation, differentiation, and antibody production (Borchers et al. 2008; Ríos 2010; Brindha 2016; Cherian et al. 2023).

This is demonstrated by APS, which promote T-cell proliferation, increases CD4^+^ and CD8^+^ T-cell counts, and modulates Th1/Th2 cytokines, often enhancing IL-2 and IFN-γ while maintaining or moderately increasing IL-4 and IL-10 (Cho and Leung 2007; Yang et al. 2013). APS also promote B-cell activation and antigen-specific IgG and IgA production, improving vaccine responses and host defense in infection models (Cho and Leung 2007; Yang et al. 2013; Yan et al. 2022b). Likewise, Echinacea polysaccharides and alkamides stimulate T- and B-cell activity and can increase Ig levels in some experimental systems, likely via TLR and cannabinoid receptor-mediated modulation of NF-κB and related transcription factors (Hudson and Vimalanathan 2011; Dapas et al. 2014).

Furthermore, Curcumin downregulates NF-κB, AP-1, and JAK/STAT signaling in T cells and antigen-presenting cells, leading to reduced IL-2, IFN-γ, and IL-17 production and limiting T-cell proliferation in hyperinflammatory or autoimmune settings (Aggarwal and Harikumar 2009; Gupta et al. 2011; Ghoushi et al. 2024). It can simultaneously promote Treg function and IL-10 expression and inhibit B-cell antibody production, thereby restraining autoantibody generation (Gupta et al. 2011; Ghoushi et al. 2024).

Other representative example is resveratrol which affects T-cell differentiation through SIRT1 and NF-κB, decreasing Th1 and Th17 responses (IFN-γ, IL-17) while enhancing Treg activity and reducing B-cell activation and autoantibody production (Minagawa et al. 2015; Shakoor et al. 2021).

Alongside this, EGCG from green tea modulates both T and B cells via antioxidant and anti-inflammatory mechanisms, including activation of Nrf2 and inhibition of NF-κB, leading to reduced Th1/Th17 cytokines and B-cell antibody secretion and increased Treg markers (Singh et al. 2010; Shakoor et al. 2021).

These actions show that phytocompounds can either enhance antigen-specific responses (e.g., Astragalus, Echinacea) or downregulate pathogenic T/B-cell responses in autoimmunity (curcumin, resveratrol, EGCG), illustrating context-dependent immunomodulation (Ríos 2010; Brindha 2016).

### Cytokine networks and Th1/Th2/Th17/Treg balance

Cytokines orchestrate immune responses, and many natural compounds exert their effects by reshaping Th1/Th2/Th17/Treg equilibrium and associated cytokine networks (Aggarwal and Harikumar 2009; Singh et al. 2010; Minagawa et al. 2015).

Several phytocompounds, including curcumin, resveratrol, and EGCG, have been shown to modulate Th1/Th2/Th17/Treg polarization and cytokine networks through coordinated regulation of NF-κB, JAK/STAT, SIRT1, and Nrf2-associated pathways, thereby promoting immune homeostasis in inflammatory and autoimmune conditions (Singh et al. 2010; Shakoor et al. 2021; Ghoushi et al. 2024). Additional phytocompounds, including andrographolide and ginsenosides, further contribute to cytokine regulation and helper T-cell polarization, supporting balanced immune responses across inflammatory and infectious conditions (Islam et al. 2018; Kim et al. 2022; Zeng et al. 2022).

Collectively, modulation of cytokine networks represents one of the principal mechanisms through which phytocompounds exert immunomodulatory effects. By influencing the balance between pro-inflammatory and anti-inflammatory mediators, these compounds can help limit excessive immune activation while preserving protective host responses. Such effects are particularly relevant in chronic inflammatory and autoimmune disorders, where dysregulated cytokine production contributes significantly to disease pathogenesis (Aggarwal and Harikumar 2009; Minagawa et al. 2015).

## Phytoimmunomodulators: different classes of plant metabolites as immunomodulators (pre-clinical data)

Tables 1, 2, 3, 4 and 5 summarizes pre-clinical studies on phytochemicals from different chemical classes including alkaloids, phenolics, lectins, glycosides, polysaccharides, saponins, triterpenoids, and sterols, along with their mechanisms of action. These plant-derived metabolites modulate immune function through diverse pathways, such as regulation of pro-inflammatory and anti-inflammatory cytokines production, controlling immune cell proliferation and differentiation, and modulation of signaling pathways involved in innate and adaptive immune responses for example, NF-κB/MAPK, and HIF-1α/NF-κB pathways. These effects were pre-clinically studied in animal models of autoimmune diseases including osteoarthritis (OA), inflammatory lung diseases and MS (experimental autoimmune encephalomyelitis (EAE)), as well as related cell lines particularly RAW264.7 murine macrophages.

**Table 1:** Preclinical studies of alkaloids as plant-derived immunomodulators and their mechanisms of action

No	Plant source	Compound & dose/concentration	Experimental model	MOA	References
1	*Hydrastis Canadensis*	Berberine (25 and 50 mg/kg)	In vivo	Suppressed pro-inflammatory IL-1β, IL-6, TNF-α, MPO, and PGE_2_ in rat models of colitis. Downregulated p38 MAPK mRNA and upregulated TGF-β. Decreased Nos2 mRNA and increased Nrf2 and Hmox. Restored redox balance (↑ GSH, SOD, CAT, GPx, GR; ↓ MDA, NO). Inhibited apoptosis by reducing Bax and caspase 3.	Jia et al. (2020)
2		Berberine (10 and 30 mg/kg)	In vivo	Attenuated disease severity and progression in EAE models. Inhibited pro-inflammatory IFN-γ, TNF-α, and IL-17. Increased anti-inflammatory IL-4, IL-10, IL-27, IL-33, IL-35, and TGF-β. Promoted Th2 and Treg responses.	Tavaf et al. (2023)
3		Berberine (10 mg/kg in vivo, 200 ng/mL in vitro)	In vitro and in vivo	Directly suppressed CD4^+^ T-cell activation and proliferation, Inhibited Th1 and Th17 differentiation, and induced T cell apoptosis by downregulating Bcl2 in vitro. Modulated JAK–STAT, PI3K/AKT, and NF-κB pathways. Restrained activated CD4^+^ T pulmonary infiltration and secondary immune cell recruitment in Acute Lung Injury (ALI) model. Decreased IFN-γ, IL-4, IL-5 and Gata3 expression.	Ma et al. (2025)
4	*Sinomenium acutum*	Sinomenine (25 or 50 mg/kg)	In vivo	Inhibited IKKβ, NF-κB, TNF-α, IL-1β, IL-6, and iNOS levels in Rheumatoid Arthritis (RA)-induced rats. Increased IL-10, Arg-1, and Fizz1. Upregulated TIMP-1 and TIMP-3 and downregulated MMP-2 and MMP-9. Modulated RANK/RANKL/OPG pathway. Activated Nrf2/Keap1 antioxidant pathway.	Li et al. (2024)
5		Sinomenine (100, 500, and 1000 µg/mL)	In vitro	Suppressed chemotaxis and secretory activity of LPS-induced macrophages. Lowered TNF-α, IL-1β, IL-6 and NF-κB p65. Inhibited TLR4/MyD88 signaling axis.	Zeng and Tong, (2020)
6	*Piper nigrum* (Black pepper)	Piperine (40 and 80 mg/kg)	In vivo	Reduced neutrophil adhesion and macrophage-derived NO and ROS in rats. Decreased IL-12 and enhanced IL-10 production. Promoted Th2 polarization via GATA3 upregulation. Inhibited Th1 and Th17 differentiation through downregulation of T-bet and RORγt.	Ghavami et al. (2024)
7	*Gelsemium elegans*	Koumine (0.5, 2.5 and 10 mg/kg)	In vivo	Reduced serum liver injury markers (ALT, AST), TNF-α and IL-6 levels in murine autoimmune hepatitis model. Upregulated Nrf2 and HO-1, downregulated Keap1. Suppressed NF-κB–mediated inflammatory signaling.	Que et al. (2023)
8		Koumine (0.4, 2, 10 and 20 mg/kg for in vivo model, 10, 40, and 70 µmol/L for in vitro analysis)	In vitro and in vivo	Inhibited B-cell activation, proliferation, and differentiation under both T cell-dependent (TD) and T cell-independent (TI) responses in vitro. Attenuated antibody (IgM and IgG1) and autoantibody secretion in primary and secondary humoral immune responses in RA mice model.	Lin et al. (2022)
9	*Stephania tetrandra*	Tetrandrine (40 mg/kg)	In vivo	Potentiated immunosuppressive Treg cell function in mice models of arthritis. Facilitated Treg cell differentiation by promoting fatty acid oxidation. Inhibited effT cell activation and proliferation. Increased IL-10 levels.	Fang et al. (2022)
10	*Sophora flavescens*	Matrine (0.5, 1.0, and 2.0 mg/mL)	In vitro	Promoted DC activation by increasing CD86 and CD83. Enhanced T cell proliferation and CTL–mediated anti-tumor activity against human gastric carcinoma cells in vitro. Increased IFN-γ, TNF-α and IL-12p70.	Zhou et al. (2022)
11		Matrine (150 mg/kg)	In vivo	Attenuated CNS autoimmunity and disease severity in EAE. Modulated IFN-β/IL-27/IL-10 pathway.	Chu et al. (2020)
12		Matrine (100 mg/kg)	In vivo	Alleviated sepsis-induced lung injury in mice. Promoted M2 and suppressed M1 macrophages. Reversed sepsis-induced activation of caspase-3 and TUNEL-positive cells, as well as antiapoptotic proteins inhibition. Modulated SIRT1/NF-κB and SIRT1/p53 pathways.	Yang et al. (2023)
13		Oxymatrine (25, 50, and 100 mg/kg)	In vivo	Reduced liver cysts, inflammation, and fibrosis in alveolar echinococcosis mouse models. Elevated hepatic CD8^+^ T-cell counts. Improved gut microbiota diversity.	Zhu et al. (2025)
14		Oxymatrine (50 mg/kg)	In vivo	Inhibited melanoma B16 cell proliferation and migration and induced apoptosis. Remodeled tumor immune microenvironment by increasing CD4^+^ T, CD8^+^ T, and NKT cells and reducing Treg cells. Enhanced TNF-α secretion and suppressed MYC and PD-L1 expression.	Li et al. (2023a)
15		Oxymatrine (0.5, 1, and 2 mg/mL)	In vitro and in vivo	Prolonged cardiac allograft survival and attenuated graft pathology in mice. Restrained splenocyte proliferation in vitro, inhibited DCs maturation and function and increased Treg and Breg cells both in vitro and in vivo. Reduced intragraft CD3^+^ and enhanced Foxp3^+^ cell infiltration.	Lan et al. (2021)
16		Oxymatrine (2.5, 5, and 10 µg/mL)	In vitro	Inhibited H9N2 avian influenza virus replication in pulmonary microvascular endothelial cells (PMVECs). Modulated TLR3 signaling, regulating downstream NF-κB and IRF-3. Regulated IFN-α, IFN-β, TNF-α and IL-6. Inhibited antiviral effector proteins PKR and Mx1.	Zhi et al. (2024)
17	Capsicum (red pepper)	Capsaicin (1.28 mg/kg)	In vivo	Reversed chronic stress–induced immunosuppression in mice. Enhanced DTH response, splenocyte proliferation and mitigated stress-induced splenocyte apoptosis. Increased Th1 and decreased Th2 cytokines and TGF-β1. Corrected corticosterone-induced increase in IL-10, IL-4, and TGF-β1.	Viveros-Paredes et al. (2021)
18	*Tinospora crispa*	Magnoflorine (25, 50 and 100 mg/kg)	In vivo	Enhanced phagocytosis, MPO activity, lysozyme levels, and NO production. Increasing CD4^+^ and CD8^+^ T-cell populations and T- and B-lymphocyte proliferation. Upregulated Th1 (IFN-γ, TNF-α, IL-2) and Th2 (IL-4, IL-6) cytokines. Elevated IgG, IgM, and DTH response.	Ahmad et al. (2022)
19		Magnoflorine (50 µM)	In vitro	Enhanced macrophage activation in LPS-stimulated U937 cells. Upregulated TNF-α, IL-1β, PGE_2_, and COX-2 expression. Activated NF-κB signaling, promoting IKKα/β, IκBα, and p65 phosphorylation and IκBα degradation. Stimulated MAPK (ERK, JNK, p38) and PI3K/AKT pathways. Upregulated TLR4/MyD88 signaling.	Haque et al. (2018)
20	*Chelidonium majus*	Chelerythrine (2.5, 5, 10, and 20 µM)	*Ex vivo and in vivo*	Blocked IL-2/IL-2Rα signaling, preventing naïve CD4^+^ T cells differentiation into CD4^+^CD25^+^Foxp3^+^ Treg cells. Downregulated Foxp3 and upregulated IFN-γ. Synergistic antitumor effect with PD-1 inhibitor in murine melanoma models.	Cho et al. (2023)
21	*Peganum harmala*	Harmine (5 and 10 mg/kg)	In vitro and in vivo	Mitigated titanium (Ti) particle-induced osteolysis in mice. Shifted macrophage polarization from M1 to M2 phenotype both in vivo and in vitro (RAW264.7 cells). Lowered TNF-α and IL-6, increased IL-4 and IL-10.	Wang et al. (2021)
22		Harmine (1, 5, 10, or 25 µmol/L for in vitro study, 50 mg/kg for in vivo study)	In vitro and in vivo	Suppressed M1 macrophage polarization, iNOS and COX-2 in LPS-stimulated macrophages. Reduced IL-1β, IL-6, TNF-α, PGE_2_, TXB2, IκBα, NF-κB and Inhibited STAT1/STAT3, NF-κB, and MAPK pathways in RAW 264.7 cells. Ameliorated murine sepsis-induced cardiac dysfunction by repressing inflammation and apoptosis. Lowered NO, IL-1β, TNF-α, NF-κB, MCP-1, NLRP3 and caspase 3 in vivo.	Ruan et al. (2022)
23	*Lycoris*	Lycorine (0.05 µM)	In vivo	Attenuated airway inflammation and remodeling in OVA-induced allergic asthma mice. Reduced total and differential immune cell counts, IgE levels, Th2 cytokines (IL-4, IL-5, IL-13), TNF-α, and oxidative stress markers (MDA, NO_2_^–^, NO_3_^–^). Augmented SOD, CAT, and GSH activities and increased IFN-γ levels.	Wang et al. (2023)
24		Lycorine (20 and 40 mg/kg)	In vivo	Ameliorated inflammation in ALI mice model. Inhibited HMGB1/TLRs/NF-κB signaling pathway. Lowered TNF-α, IL-1β, IL-6 in BALF and LPS-stimulated lung cells.	Ge et al. (2020)
25					
26	Tobacco	Nicotine (10 µM)	In vitro	Downregulated TLR-2, TLR-4, and NOD-2, in Mtb-infected lung epithelial cells and macrophages. Reduced SP-D in type II pneumocytes. Inhibited IL-6, CCL5 in epithelial cells, and IL-8, IL-6, TNF-α, IL-10, CCL2, CXCL9, CXCL10 in macrophages.	Valdez-Miramontes et al. (2020)

**Table 2:** Preclinical studies of polyphenols as plant-derived immunomodulators and their mechanisms of action

No	Plant source	Compound & dose/concentration	Experimental model	MOA	References
1	Chinese lacquer tree	Butein (10, 20, and 40 mg/kg)	In vivo	Suppressed TWEAK‑FN14 signaling in cSCC models. Inhibited NF‑κB and STAT3 inflammatory pathways. Reduced IL-1β, IL-6, TNF-α and IFN-γ.	Dong et al. (2025)
2		Butein (50 and 100 mg/kg)	In vivo	Preserved liver integrity and limited cell damage in 5-FU-induced rat model of hepatotoxicity. Decreased liver enzymes, MDA, IL-6, NF-κB, TNF-α, and caspase-3 while increasing IL-10, GSH, and NRF2.	Mohammed and Al-Shawi (2025)
3	*Lithospermum erythrorhizon* and *Arnebia Euchroma*	Shikonin (20 mg/kg/day)	In vivo	Ameliorated EAE in mouse model. Reduced TNF-α and IFN-γ in CNS tissue. Upregulated TGF-β and Bcl-2. Increased GPX-1 activity.	Nasrollahzadeh Sabet et al. (2020)
4		Shikonin (8 mg/kg)	In vivo	Attenuated sepsis-induced immunosuppression in mice models. Inhibited PKM2-dependent metabolic and transcriptional signaling in macrophages and suppressed PD-L1. Restored T-cell immune response. Inhibited IL-1β, IL-6, TNF-α, and IFN-γ.	Yuan et al. (2023)
5	*Curcuma longa*	Curcumin (20 mg/mL for in *vivo* study, 100 ng/ml for In vitro study)	In vitro and in vivo	Mitigated lung injury in ALI animal models. Suppressed IL‑17 A, NF‑κB p65, and MPO^+^ neutrophils in lung tissue. Enhanced Treg differentiation and IL‑10 levels in vivo and in vitro (naïve CD4^+^T cells).	Chai et al. (2020)
6		Curcumin (4.33 mg/kg)	In vivo	Modulated innate immune response in immunosuppressed, *Cryptosporidium parvum*–infected neonatal mice. Downregulated IFN-γ and IL-18. Regulated gut microbiota composition.	Rahman et al. (2022)
7	*Iris halophila*	Piceatannol (2 µM for In vitro assay, 10, 20, 40 mg/kg for In vivo assay)	In vitro and in vivo	Restrained maturation and function of DCs, in vitro and in vivo by inhibiting MAPK and NF‑κB signaling (immune suppression). Decreased CD4^+^ T‑cell polarization (Th1 and Th17), elevated Th2, iTreg and nTreg cells in experimental arthritis model. Reduced TNF‑α, IL‑6, IL‑17 A, IFN‑γ and increased IL‑10.	Han et al. (2025)
8	*Scutellaria baicalensis*	Wogonin (50 µg/ml)	In vivo	Shifted macrophage polarization from M1 to M2 in mouse OA models. Suppressed LSD1-mediated NF-κB p65 activation. Lowered IL-6, TNF-α and IκBα.	Cai et al. (2025)
9	*Passiflora incarnata*	Chrysin (12.5, 25 and 50 mg/kg)	In vivo	Reversed DZN-induced depletion of CD4^+^T cells, CD8^+^T cells and NK, and enhanced phagocytosis and DTH responses in mice. Increased specific anti-SRBC, total IgM/IgG, T-bet and IFN-γ. Corrected DZN-induced imbalances in GATA3, RORγt, FOXP3 decreasing IL-4, IL-10, IL-17, and TGF-β.	Zeinali et al. (2022)
10	*Bidens pilosa*	Centaurein (75 µg/ml), Centaureidin (0.9 µg/ml)	In vitro	Upregulated IFN-γ transcription in T cells by activating NFAT and NF-κB signaling pathways.	Chang et al. (2007)
11	Blue honeysuckle berries	Cyanidin‑3‑glucoside (C3G) (25 mg/kg in vivo, 25, 50, or 100 µM in RASF culture and 50 µM in MNCs culture)	In vitro and in vivo	Ameliorated CIA, increased IL-10 and Treg cell count, and inhibited IL-6, IFN-γ and CD38 + NK cells in rats. Promoted apoptosis, suppressed proliferation of RASF and reduced IL-6 in vitro. Increased IL-2, IL-10 and Treg cells, decreased IL-6, IFN-γ and CD38 + NK cells in MNCs. Upregulated Sirt6 and downregulated NKG2D, increased TNF-α and decreased IFN-γ in co-culture CD38 + NK cells stimulating MNCs differentiation into Treg cells.	Wang et al. (2019)
12		C3G (50 µM)	In vitro	Inhibited IL-1β and NF-κB phosphorylation in LPS-stimulated MSCs. C3G-treated MSC-conditioned medium suppressed pro-inflammatory cytokines (IL-1β, IL-6, IL-12, TNF-α in macrophages; IL-2 in lymphocytes) and enhanced anti-inflammatory IL-10 in both cell types, primarily via NF-κB pathway inhibition.	de Freitas et al. (2025)
13		C3G (100, 200, and 400 mg/kg)	In vivo	Attenuated silica-induced lung inflammation in mice by reducing lung inflammatory cell infiltration and MCP-1, TNF-α, and in BALF. Suppressed CD4^+^ and CD8^+^ T-cell–associated cytokine production. Decreased Tregs and IL-17 A–producing Treg populations in lung tissue. Blocked STAT1/STAT3 signaling in lung.	Zhao et al. (2020)
14	*Polygonum orientale*	Orientin (10 and 20 mg/kg in vivo, 10 and 30 µM in vitro)	In vitro and in vivo	Mitigated lung injury, enhanced survival, and reduced inflammatory cytokines in murine sepsis models via direct inhibition of PFKL. Decreased LPS-induced pro-inflammatory cytokine expression in BMDMs and RAW264.7 cells. Suppressed macrophage glycolysis (↓ lactate production, ↓ glucose uptake, ↓ ECAR).	Zheng et al. (2026)
15		Orientin (10, 20, and 40 mg/kg)	In vitro and in vivo	Alleviated skin lesions, keratinocyte hyperproliferation and immune cell infiltration in psoriasis mouse model induced by IMQ. Suppressed IL-6, IL-8, IL-17, IL-23, IL-1β and TNF-α in vivo and in LPS-induced RAW264.7 and HaCaT cells. Reversed the increase in ROS and the decrease in IL-10 and glutathione induced by LPS. Downregulated MAPK signaling.	Long et al. (2024)
16		Orientin (50 mg/kg for in vivo study, 2, 8, or 32 µM for in vitro study)	In vitro and in vivo	Inhibited microglial M1 polarization, suppressed TNF-α, IL-1β and IL-6, and elevated IL-10 in LPS-stimulated BV2 cells and mice. Attenuated neuroinflammation in PFC in LPS- and CUMS-induced depression murine models through modulation of PI3K/AKT signaling pathway.	Du et al. (2025)
17	*Crataegus spp.* (Hawthorn), *Vitex spp.*, and *Passiflora spp.*	Vitexin (15 mg/kg and 30 mg/kg)	In vivo	Reduced IgE, Th1/Th2/Th17 responses, IL-4, IFN-γ, chemokines, MMP-1, MMP-13, and oxidative mediators in OVA-LPS induced allergic asthma mice model. Increased IL-10, IL-15, and modulated MMP-9, MMP-3. Inhibited TGF-β/Smad2/3/4, Caspase-9/3 and restored autophagy balance (↓ LC3A/B; ↑ Beclin-1, p62; normalized Bcl-2/Bax). Downregulated NF-κB, PI3K/AKT, p38 MAPK, STAT3, GATA3 and restored PTEN expression.	Tirpude et al. (2022)
18		Vitexin (20 and 40 mg/kg in vivo, 5 and 10 µM in vitro)	In vitro and in vivo	Reprogrammed TAMs toward an M1 (iNOS^+^) phenotype and enhanced CD8^+^ T-cell antitumor immunity in mouse model of allograft breast tumor. Promoted M1 macrophage polarization in vitro (with LPS + IFN-γ).	Chen et al. (2022d)
19		Vitexin (40 mg/kg)	In vitro and in vivo	Reduced proliferation and metastatic potential of TNBC cells and increased TNF-α and IL-1β in vitro. Enhanced M1 and decreased M2 macrophage polarization. Inhibited EGFR phosphorylation and downstream PI3K/AKT/mTOR signaling. Limited tumor growth, shifted TAM balance toward M1 phenotype, and exhibited a synergism with doxorubicin in vivo.	Lin et al. (2024)
20		Vitexin (40 mg/kg)	In vivo	Restored Th17/Treg balance, Reduced IL-18, IL-6, IL-17 and increased IL-10 in OVA-induced asthmatic mice. Downregulated Notch signaling pathway (↓ Notch1, DLL4, Hes1 expression in lung tissue).	Fei et al. (2024)
21		Vitexin (40 mg/kg)	In vivo	Lowered endothelial chemokines CXCL1 and CX3CL1 in brain tissue of SE-induced mice. Suppressed leukocyte-endothelial adhesion by modulating ICAM-1, VCAM-1, and E-selectin. Reduced IL-6, IL-8, TNF-α, NF-κB p65 and MCP-1, and increased IL-10.	Cao et al. (2020)
22		Vitexin (10 mg/kg)	In vivo	Ameliorated arthritic alterations in CIA rat model. Decreased IL-1β, IL-6, IL-17, TNF-α, IFN-γ, NO and iNOS and normalized IL-4 and IL-10. Downregulated JAK/STAT signaling and upregulated SOCS expression.	Zhang et al. (2022a)
23		Vitexin (10, 20 or 40 µM in vitro, 15, 30, and 60 mg/kg in vivo)	In vitro and in vivo	Activated PPARγ, decreasing ROS production and increasing SOD, GSH-Px, and CAT in S. aureus-infected bovine mammary epithelial cells and S. aureus-induced mastitis animal model. Suppressed TNF-α, IL-1β, IL-6 and apoptosis both in vitro and *in vivo.* Attenuated ER stress–driven MAPK/NF-κB inflammatory signaling.	Chen et al. (2022c)
24	Hawthorn	Vitexin-2-*O*-rhamnoside (100, and 200 mg/kg)	In vivo	Restored immune organ function in CY-immunosuppressed mice. Promoted proliferation and activity of T and B lymphocytes, NK cells, and CTLs. Upregulated IFN-γ, IL-2, IL-6, and IL-12. Stimulated PI3K/AKT signaling cascade. Elevated GSH-Px, CAT, SOD, and T-AOC and reduced MDA levels.	Wang et al. (2022b)
25	Onions, blueberries, green tea	Quercetin (10, 20, 40 and 80 µM in vitro, in vivo)	In vitro and in vivo	Reduced tumor immune evasion through inhibition of 4T1 cell proliferation, migration, and invasion via Suppressing IL-6/JAK2/STAT3 signaling. Depleted Treg cells within tumor microenvironment and enhanced cytotoxic anti-tumor immunity in 4T1 xenograft mice.	Liao et al. (2024)
26		Quercetin (100 mg/kg)	In vivo	Modulated the immunoregulatory index (CD4+/CD8+), indicating regulation of cell-mediated immunity in experimental bacterial-immune periodontitis in rats. Modulated adaptive immunity (CD3+, CD4+, CD8+, CD19+, CD16+) and restored T-cell function. Normalized B-cell and NK cell populations.	Demkovych et al. (2021)
27		Quercetin (20 and 50 mg/kg in vivo, 20 µM in vitro)	In vitro and in vivo	Inhibited monocyte-to-macrophage differentiation, macrophage polarization, and mast cell activation in vitro (BMDMs). Suppressed immune cell recruitment and attenuated inflammatory responses during HO progression in vivo (murine burn/tenotomy model). Activated SIRT1 and reduced acetylated NF-κB p65 via modulation of the SIRT1/NF-κB signaling pathway both in vitro and in vivo.	Li et al. (2021)
28	*Terminalia chebula*	Chebulagic acid (100 and 200 mg/kg)	In vivo	Inhibited RIG-I–dependent NF-κB, p38/JNK/ERK MAPK, and JAK/STAT signaling in H1N1-induced ALI mice models. Targeted IDO1–Kyn axis, reduced Kyn/Trp ratio, and restored Trp metabolic homeostasis. Blocked IDO1-Kyn–mediated AhR activation and IFN-β–driven pro-inflammatory feedback loops.	Li et al. (2025b)
29		Chebulagic acid	In vitro and in vivo	Promoted anti-tumor immunity, reducing tumor growth and increasing apoptosis in vivo. Induced apoptosis and suppressed LUAD cell proliferation in vitro and in vivo. Enhanced NK cell migration and infiltration via FBXO38/CCL5 signaling.	Hu et al. (2026)
30	*Caesalpinia coriaria*	Corilagin (25 mg/kg)	In vivo	Suppressed M1 macrophage activation in Con A-induced hepatitis murine model. Reduced IL-6, IL-12, and iNOS by modulating the MAPK, NF-κB, and IRF signaling pathways, limiting hepatocyte apoptosis and oxidative stress.	Yan et al. (2022a)
31		Corilagin (12.5, 25, or 50 µg/mL in vitro, 40 mg/kg in vivo)	In vitro and in vivo	Ameliorated HSE by interfering with the TLR3 signaling pathway both in vitro and in mice models. Suppressed the expression of downstream mediators including TRIF, TRADD, TRAF3/6, NEMO, P38, and IRF3. Reduced IL-6, TNF-α, and IFN-β.	Li et al. (2019)
32		Corilagin (60, 120 and 240 µM in vitro, 15, 30, and 60 mg/kg in vivo)	In vitro and in vivo	Inhibited M1 macrophage polarization (decreased iNOS, increased CD206) in atherosclerotic mice and LPS-stimulated RAW264.7 macrophages. Lowered TNF-α, IL-1β, IL-6, and IL-18 levels. Downregulated TLR4–NF-κB/MAPK signaling.	Meng et al. (2023)
33		Corilagin (IC50 = 41.25 µmol/L)	In vitro	Restrained macrophage proliferation and foam cell formation in LPS-induced RAW264.7 cells. Downregulated CD40–CD40L–TRAF-1 signaling and reduced IL-6 and IFN-γ. Modulated macrophage activation and lipid accumulation via suppression of the CD40–CD40L axis.	Zhang et al. (2025)
34	Pomegranate	Punicalagin (12.5, 25, and 50 mg/kg)	In vivo	Inhibited NF-κB and JAK2/STAT3 signaling pathways in mouse model of spondylitis. Decreased serum TNF-α, IL-1β, IL-6, IL-17 A, and IL-23. Regulated Th17 cell response and IL-17 A/IL-23 axis. Reduced ROS and lipid peroxidation, and enhanced CAT, GPx and SOD enzymes.	Feng et al. (2020)
35		Punicalagin	In vitro and in vivo	Enhanced phagocytic capacity of macrophages by upregulating the expression of Clec4e. Activated NF-κB and MAPK signaling pathways improving bacterial clearance in mouse models challenged with E. coli and P. aeruginosa.	Yin et al. (2024)
36		Punicalagin (12.5, 25, and 50 mg/kg)	In vivo	Decreased inflammatory cell infiltration in BALF and suppressed Th2-mediated airway inflammation in OVA-induced asthma murine model. Reduced Th2 cytokines (IL-4, IL-5, and IL-13) and OVA-specific IgE levels. Modulated IL-4/STAT6 and Notch/GATA3 signaling pathways.	Yu and Li (2022)
37	*Mangifera indica*	Mangiferin (50 and 100 mg/kg)	In vivo	Restored liver architecture and function in MTX-treated rats. Activated Nrf2 signaling, reducing oxidative stress (↑ glutathione, ↓ MDA). Suppressed NF-κB/NLRP3 inflammasome axis.	El-Sayed et al. (2025)
38		Mangiferin (30 and 100 µM)	In vitro	Inhibited T lymphocyte proliferation in a dose-dependent manner. Reduced oxidative stress by lowering MDA, NO and MDA/GSH ratio, while increasing GSH. Decreased IFN-γ and IL-4, increased IL-10. Stronger anti-inflammatory and immunomodulatory effects than dexamethasone.	Askari et al. (2025)
39		Mangiferin (50 mg/kg)	In vitro and in vivo	Reduced inflammatory cell infiltration via inhibiting Th2-cytokines, histamine, TSLP, LTB4, and IgE levels in atopic dermatitis mouse model. Inhibited MAPK and NF-κB phosphorylation in macrophages.	Lu et al. (2024)
40		Mangiferin (5, 10, and 25 µM)	In vitro	Activated Nrf2, increased HO-1/CAT, and suppressed NF-κB, COX-2, IL-6 in hyperglycemia-induced macrophages. Inhibited NLRP3 inflammasome and ROS accumulation. Restored AKT phosphorylation and enhanced macrophage migration and invasiveness, promoting wound healing.	Jayasuriya et al. (2024)
41		Mangiferin (10, 50, or 100 µg/mL)	In vitro	Alleviated LPS- induced BMDMs pyroptosis by Inhibition of pyroptosis-related genes and proteins: Caspase-1, Caspase-11, NLRP3, GSDMD and NF-κB. Downregulated TNF-α, IL-1β, IL-6, and IL-18 and suppressed NF-κB signaling.	Feng et al. (2022)
42		Mangiferin (50, 100, 150 or 200 µmol/L)	In vitro	Exerted immunosuppressive action by increasing Bregs and IL-10 via JAK2/STAT3 and ERK signaling in murine splenic MNCs. Decreased IL-2 and IFN-γ expression. Activated Nrf2/NQO1 antioxidant pathway and inhibited Keap1.	Qin et al. (2021)
43	–	Luteolin (2 µM in vitro, 0.2 mg/kg in vivo)	In vitro and in vivo	Downregulated IL-17 A, MPO, and NF-κB in ALI murine models. Increased CD4^+^CD25^+^FOXP3^+^ Treg frequency and upregulated IL-10 levels in serum, BALF, PBMCs, and splenic MNCs. Promoted M2 and reduced M1 macrophage polarization. Induced Treg differentiation from naïve CD4^+^ T cells and IL-10–mediated M2 macrophage polarization in vitro (RAW 264.7).	Xie et al. (2021)
44		Luteolin (0.1 and 1 mg/kg)	In vivo	Mitigated nasal symptoms and mucosal inflammation in OVA-induced Allergic rhinitis rat model. Downregulated Th2 cytokines (IL-4, IL-5, IL-13) and OVA-sIgE, and partially normalized Th1 cytokines (IFN-γ, IL-2). Suppressed TLR4/NF-κB (p65) signaling in nasal mucosa and restored Th1/Th2 balance.	Dong et al. (2021)
45		Luteolin (5 and 10 mg/kg)	In vivo	Reduced Th2 cytokines (IL-4, IL-5, IL-13) and improved olfactory function in ECRS mice in vivo. Suppressed TLR4/NF-κB signaling and P65 nuclear translocation both in vivo and in HOEPCs in vitro. Decreased OSN apoptosis (cleaved caspase-3/9, Bcl-2) and oxidative stress (SOD, CAT, GSH-Px, MDA).	Shi et al. (2025)
46					
47	*Scutellariae radix*	Baicalein (5 and 10 mg/kg)	In vivo	Reduced abortion rates and improved embryonic development in URSA murine model. Expanded Treg cells and increased decidualization markers (IGFBP-1, PRL). Suppressed TLR4/NF-κB signaling (↓ TLR4, p-P65, P65) at the maternal-fetal interface.	Huang et al. (2025a)
48		Baicalein (10, 20, and 40 µM)	In vitro	Enhanced anti-tumor immunity in TNBC by suppressing adipocyte-derived leptin, reducing PD-L1 expression, and inhibiting p-STAT3 signaling in the tumor microenvironment.	Liu et al. (2023)
49		Baicalein (50 µM)	In vitro	Provoked CD274/PD-L1 autophagic degradation via LC3, boosting T-cell–mediated antitumor immunity and inhibiting tumor growth.	Hao et al. (2025)
50		Baicalein (25 µg/mL)	In vitro	Protected BMECs from apoptosis and tight junction injury in vitro and reduced neurological deficits and immune cell infiltration in rat ischemia-reperfusion (IR) model. Suppressed TNF-α, IL-6, and iNOS, and inhibited TLR2/4–NF-κB signaling.	Hao et al. (2023)
51		Baicalein (25, 50, and 100 µM)	In vitro	Inhibited Mtb-induced macrophage pyroptosis by suppressing AIM2/NLRP3 inflammasome assembly and IL-1β release. Promoted autophagy via Akt/mTOR pathway inhibition, supporting host-directed therapy against tuberculosis (TB).	Ning et al. (2023)
52		Baicalein (20 mg/kg)	In vivo	Improved survival, reduced bacterial load and organ damage, restored CD4^+^ T cells and CD4^+^/CD8^+^ ratios, and decreased leukocyte apoptosis in rat model of sepsis. Suppressed TNF-α and IL-6, increased IL-10 and galectin-9 expression.	Chen et al. (2022a)
53		Baicalein (50 mg/kg)	In vivo	Inhibited tumor growth in breast cancer and melanoma models by promoting TAM infiltration and skewing macrophages toward an M1-like phenotype, increasing TNF-α, IL-1β, CXCL9, and CXCL10. Inhibited PI3Kγ, activating NF-κB/TNF-α signaling to drive M1 polarization and enhance antitumor immunity.	He et al. (2021)
54	–	Resveratrol (100 µmol/L)	In vitro	Disrupted PD-L1 glycosylation and stability, reducing its surface expression by targeting glyco-processing enzymes and binding the PD-1 interaction interface. Enhanced CTL activity by inhibiting the PD-1/PD-L1 immune checkpoint.	Verdura et al. (2020)
55	Ginger	Gingerol (100 mg/mL, 200 µl/day)	In vivo	Increased survival and alleviated clinical symptoms of cGVHD in MHC-mismatched models. Decreased Th1/Th17 (TNF-α^+^, IFN-γ^+^, IL-17^+^) T cells and increased Foxp3^+^CD4^+^ Tregs. Acted as a P2RY13 antagonist, reprogramming T-cell responses via CD244/PD-1 induction and modulation of calcium signaling and glycolysis to constrain T-cell activation.	He et al. (2025)
56		6-gingerol (2 µM in vitro, 0.1, 1 or 2.5 mg/kg in vivo)	In vitro and in vivo	Inhibited viral neuraminidase activity and reduced H1N1 infection in MDCK cells. Lowered lung viral load and reduced mortality in H1N1-infected mice. Suppressed neuraminidase-mediated TGF-β activation in lung CD4 + T cells and enhanced hemagglutinin-specific Th1 and Th17 responses.	Dutta et al. (2023)
57		6-gingerol (10 mg/kg)	In vivo	Increased CD8 + T cell percentage and upregulated CTLA-4 in AOM/DSS-induced colorectal cancer mice. Decreased PD-1, B7, and PD-L1. Inhibited immune checkpoint signaling and enhanced CD8 + T cell function via PD-1/PD-L1 and CTLA-4/B7 pathways.	Ajayi and Adeshina (2022)
58		6-gingerol (50 nM)	In vitro	Activated TRPV1 in primary human neutrophils, increased surface expression of CD11b, CD66b, and FPR1, enhanced CXCL8 secretion and ROS production upon fMLF stimulation, and thereby facilitated canonical cellular immune responses.	Andersen et al. (2023)
59		6-gingerol (100 and 250 mg/kg)	In vivo	Reduced DSS-induced weight loss and bowel tissue damage in mice. Decreased IL-17 and increased IL-10 levels in serum and colon. Suppressed NF-κB signaling by inhibiting IκBα and p65 phosphorylation.	Sheng et al. (2020b)
60		6-gingerol (50 µg)	In vitro	Promoted bacterial clearance in septic mice models. Inhibited TNF-α and IL-6 production in macrophages stimulated via TLR2 and TLR4. Enhanced neutrophil count and function at infection sites and reduced immune cells apoptosis. Shifted Th2/Th1 cytokine balance toward Th1.	Ju et al. (2021)
61		6-gingerol (100 and 250 mg/kg)	In vivo	Alleviated bowel tissue damage in DSS-induced colitis mice. Reversed DSS-induced increase in IL-6 and IL-17 and decrease in IL-10 levels. Suppressed Th17 cell expansion and restored Treg cell count in blood. Downregulated RORγT and upregulated FOXP3 expression, restoring Th17/Treg balance.	Sheng et al. (2020a)
62	*Ginkgo biloba*	Apigenin-7-O-glucoside (25 and 50 mg/kg)	In vivo	Ameliorated DSS-induced colitis by improving colon length and histopathology in mice. Normalized inflammatory mediators and modulated PPARγ expression. Strengthened intestinal barrier by upregulating ZO-1, occludin, claudin-1, and claudin-3. Suppressed MAPK signaling pathways including ERK, JNK, and p38. Downregulated COX-2 and iNOS expression. Modulated gut microbiota.	Hu et al. (2023b)

**Table 3:** Preclinical studies of lectins and glycosides as plant-derived immunomodulators and their mechanisms of action

No	Plant source	Compound & dose/concentration	Experimental model	MOA	References
1	*Artocarpus heterophyllus*	ArtinM (0.5 µg/animal, Adjuvant)	In vivo	Minimized fungal burden and tissue damage in lungs of *Cryptococcus gattii* infected mice. Increased IL-10 levels and reduced IL-23 and iNOS.	Martins Oliveira-Brito et al. (2024)
2	Jack bean	Concanavalin-A (250 µg)	In vitro	Mitigated parasitemia in mice by promoting M1 macrophage polarization. Stimulated IL-17 A and NO production via cNOS and iNOS upregulation. Elevated TNF-α and IL-6 upon infection.	Zanluqui et al. (2020)
3	Jackfruit seeds	Jacalin (200 µg/mL for 6, 12, 24, and 48 h)	In vitro	Stimulated PBMC proliferation. Enhanced early pro-inflammatory signaling (elevated IFN-γ). Elevated anti-inflammatory TGF-β expression on prolonged exposure.	Lavanya et al. (2022)
4	*Rheum palmatum*	Emodin-8-O-glucoside (1, 2.5, 5, 10 and 20 µM)	In vitro	Upregulated TLR-2/MAPK/NF-κB pathway in murine RAW264.7 macrophages. Induced TNF-α, IL-6 secretion, stimulated iNOS and increased NO.	Lee et al. (2020)
5	*Allium sativum* (garlic)	Rutin (40 mg/kg)	In vivo	Alleviated immunopathology in murine schistosomiasis model. Restored Th1/Th2 balance, suppressed Th17-associated inflammation. Reduced TNF-α, IL-17, IL-4 and hepatic granuloma formation.	Hamad (2023)
6	Citrus fruits	Hesperidin (5, 10 and 20 mg/kg)	In vivo	Relieved symptoms of allergic rhinitis in mice models. Lowered histamine, NO, IL-4, IFN-γ/IL-4 ratio, IL-6, IL-10, IgE, total protein and PLA_2_. Downregulated ROR-γt, GATA-3 and upregulated FOXP-3 and T-bet.	Liu et al. (2022)
7	*Aucuba japonica*	Aucubin (2.5, 5, 10, and 25 µM in vitro, 10 mg/kg in vivo)	In vitro and in vivo	Reduced LPS-induced NO, TNF-α, and IL-6 production in RAW 264.7 cells. Decreased IL-4, IL-5, IL-13, TNF-α, IgE, inflammatory cell infiltration, and oxidative stress markers (MDA, MPO, NO). Increased IFN-γ, antioxidant levels, and alleviated airway inflammation in OVA-induced asthmatic mice.	Li et al. (2025a)
8		Aucubin (50 and 100 µM in vitro, 5, 10 and 40 mg/kg in vivo)	In vitro and in vivo	Suppressed LPS-induced microglial activation and pro-inflammatory cytokine release by downregulating NF-κB and MAPK signaling pathways in primary microglia. Attenuated ischemic injury and neuroinflammation via suppression of NF-κB and MAPK pathways in MCAO mice.	Liang et al. (2024)
9		Aucubin (20, 40, 80 µmol/L in vitro, 6 mg/kg in vivo)	In vitro and in vivo	Alleviated joint inflammation and bone destruction, decreased inflammatory factor expression, and suppressed NF-κB pathway activation in CIA rat model. Downregulated NF-κB signaling by inhibiting p-IκKα/β, p-IκBα, and p-p65, as well as NF-κB-p65 nuclear translocation and activity in RAW264.7 cells.	Zhang et al. (2022b)

**Table 4:** Preclinical studies of polysaccharides as plant-derived immunomodulators and their mechanisms of action

No	Plant source	Compound & dose/concentration	Experimental model	MOA	References
1	Radix Astragali	APS (0, 25, 50, or 100 µg/mL)	In vitro	Activated NF‑κB p65/MAPK pathway in RAW264.7 macrophages. Increased NO production, upregulated TNF‑α, IL‑6 and iNOS.	Feng et al. (2021)
2	Korean red ginseng	Red ginseng acidic polysaccharide (RGAP) (100 mg/kg)	In vivo	Enhanced IgM antibody-forming cells and phagocytic activity in immunosuppressed mice.	Youn et al. (2020)
3	Aloe vera gel	Acemannan (50 mg/kg)	In vivo	Modulated immunity and minimized radiation-induced mortality in mice. Stimulated hematopoiesis and increased TNF-α and IL-1.	Kumar and Tiku (2016)
4	–	Pectin (10 mg/kg)	In vivo	Enhanced anti-PD-1 mAb efficacy in CRC patient–microbiota–humanized tumor-bearing mice by increasing CD8^+^ T cell infiltration and activation in tumor microenvironment; CD8^+^ T-cell depletion abrogated the anti-tumor effect. Remodeled gut microbiota by increasing diversity and enriching butyrate-producing bacteria, elevated SCFA butyrate levels, and via microbiota-dependent mechanisms (FMT) overcame anti-PD-1 resistance.	Zhang et al. (2021)
5	–	β-Glucan (10, 50, and 100 µg/mL)	In vitro	Potentiated macrophage immune function by dose-dependently inducing inflammatory mediators (PGE_2_, NO) and pro-inflammatory cytokines (TNF-α, IL-6), promoting morphological activation and phagocytic activity in vitro. Promoted keratinocyte-mediated wound healing.	Xin et al. (2022)
6	*Fructus aurantii*	CALB-4 (20, 40, and 80 µg/mL)	In vitro	Enhanced immune responses in human PBMCs by increasing cytoplasmic pro-IL-1 levels through upregulation of MAPKs and nuclear translocation of p65.	Shu et al. (2018)
7	*Garcinia mangostana*	GMP90-1 (200 µg/mL)	In vitro	Improved macrophage phagocytosis and increased NO, IL-6, IL-1β, and TNF-α secretion in vitro, and these immunomodulatory effects were confirmed in vivo in zebrafish.	Zhang et al. (2020)

**Table 5:** Preclinical studies of saponins, terpenoids and sterols as plant-derived immunomodulators and their mechanisms of action

No	Plant source	Compound & dose/concentration	Experimental model	MOA	References
1	*Panax ginseng*	Ginsenosides (50, 100, and 200 mg/kg/day)	In vivo	Upregulated TLR4/MyD88/NF-κB pathway in immunosuppressed mice.	Li et al. (2025c)
2	*Centella asiatica*	Asiaticoside (50 µM in vitro, 50 mg/kg in vivo)	In vitro and in vivo	Suppressed tumor metastasis and growth, restored NK cell function and increased IFN-γ secretion in B16 subcutaneous mice tumor model. Abrogated immunosuppressive effects induced by TGF-β in NK cells. Downregulated and destabilized TGF-β receptors (TGFBR1/TGFBR2). Inhibited SMAD2 phosphorylation and mitochondrial translocation.	Guo et al. (2024)
3		Asiaticoside (10 mg/kg)	In vitro	Potentiated antitumor immunity of anti-PD-L1 therapy in hepatocellular carcinoma mouse model. Enhanced T-cell activation via LCK/AKT signaling pathway. Increased proportion of effT cells in the spleen. Elevated TNF-α and reduced IL-6 levels.	Ren et al. (2025)
4		Asiaticoside (20, 40, and 60 mg/kg in vivo, 100 µM in vitro)	In vitro and in vivo	Protected mice against IR–induced AKI. Suppressed IL-6, IL-1β, TNF-α and increased anti-inflammatory IL-10. Shifted macrophage polarization from M1 to M2 phenotype. Attenuated LPS-induced inflammatory responses in RAW264.7 macrophages. Inhibited iNOS and promoted Arg-1.	Tang et al. (2022)
5	Glycyrrhiza	Glycyrrhizin (25 or 50 mg/kg)	In vivo	Reduced serum total IgE and mast cell infiltration in skin lesions in atopic dermatitis murine model. Decreased IL-4, IFN-γ, TNF-α, and TSLP. Inhibited Th1/Th2/Th17-driven immune responses. Suppressed LCs migration and mast cell infiltration.	Hou et al. (2022)
6	*Andrographis* *paniculata*	Andrographolide (12.5, 25, and 50 µM)	In vitro	Reduced IL-1β, IL-6 and TNF-α and blocked NF-κB pathway activation in Mtb-infected murine macrophages. Promoted autophagic degradation of NLRP3 inflammasome. Suppressed PI3K/AKT/mTOR and Notch1/Akt/NF-κB pathways. Inhibited IL-1β and modulated IL-8 and MCP-1 in co-culture epithelial cells.	He et al. (2020a)
7	*Boswellia sacra*	β-Boswellic acid (50 µM)	In vitro	Downregulated immune checkpoint molecules PD-1 and TIGIT on activated CD4^+^ and CD8^+^ T cells in vitro, reinvigorating activated/exhausted T cells.	Meyiah et al. (2025)
8		Boswellic acids	In vivo	Reduced brain demyelination, clinical severity and duration of EAE symptoms in mice. Downregulated IFN-γ, IL-17, T-bet and ROR-γt. Upregulated TGF-β, FoxP3 and GATA3.	Shadab et al. (2024)
9	*Salvia officinalis*	Ursolic acid (25 mg/kg)	In vivo	Enhanced jejunal immune homeostasis and strengthened intestinal barrier function in mice. Lowered IL-1β, IL-6, and TNF-α Modulated gut microbiota composition. Downregulated IL1R1 and CACNB2 and promoted retinol and ascorbic acid biosynthesis pathways.	Zhao et al. (2024b)
10		Ursolic acid (1 and 4 µM)	In vitro	Impaired Th17 differentiation via inhibiting STAT3/RORγt signaling pathway. Inhibited Schwann cell–mediated Th17 migration, primarily via CXCL9/10–CXCR3 axis.	Xu et al. (2022a)
11		Ursolic acid (25, 50, and 100 mg/kg)	In vivo	Reduced FBG and minimized pancreatic β-cells destruction in Streptozotocin (STZ)-induced T1DM rats. Restored gut microbiota diversity and balance. Corrected Th17/Treg cell imbalance: increased Treg markers (Foxp3, IL-10) and decreased Th17 markers (RORγt, IL-17 A, TNF-α).	Chen et al. (2022b)
12	–	β-sitosterol	In vivo	Ameliorated asthma symptoms in rat models. Reduced IgE, TNF-α, TGF-β1, IL-5, IL-6, IL-13, and IL-21 and increased IFN-γ and IL-10. Lowered macrophages and neutrophils, downregulated CD68 and MPO in spleen. Elevated Th cells, Tc cells, NK cells, and DCs in peripheral blood. Increased CD11c, CD80, and CD86 levels in spleen and lung.	Wang et al. (2022a)
13	*Withania somnifera*	Withaferin A (2 mg/kg in vivo, 0.5 µg/mL ex vivo)	Ex vivo and in vivo	Restricted intracellular drug-sensitive and drug-resistant Mtb by promoting macrophage M1 polarization and enhancing Th1/Th17 responses *ex vivo and in vivo*. Augmented effT cell function and induced memory T cell populations via STAT signaling, reducing TB reactivation/reinfection in mice model; showed adjunct efficacy with isoniazid.	Kumari et al. (2023)

## Role of immunomodulatory phytocompounds in restoration and regulation of immune function across disease states

### Protection against therapy-induced leukopenia and immunosuppression

Several herbal immunomodulators protect against or reverse leukopenia and broad immunosuppression induced by cytotoxic chemotherapy or radiation (Brindha 2016; Li et al. 2023b).

A notable example is APS which support hematopoiesis by promoting proliferation and differentiation of bone marrow progenitor cells and reducing chemotherapy-induced apoptosis, restoring total white blood cell (WBC) counts, neutrophils, and lymphocytes in cyclophosphamide-treated models (Li et al. 2023b). Additionally, *Ganoderma* β-glucans improve bone marrow recovery, increase peripheral leukocytes, and enhance NK and T-cell function in immunosuppressed animals and patients, partly via hematopoietic growth factor modulation and antioxidant effects (Borchers et al. 2008). Similarly, Ginsenosides may mitigate myelosuppression by supporting progenitor survival, improving redox status, and modulating cytokines that regulate hematopoiesis, thereby improving leukocyte recovery and immune competence (Kim et al. 2022; Lee et al. 2025).

Formulations combining mushrooms, Astragalus, ginseng, and other botanicals can synergistically restore total white cell counts, neutrophils, lymphocytes, and NK activity in chemotherapy-induced immunosuppression and other iatrogenic immunodeficiency states (Kim et al. 2022; Zheng et al. 2023). Mechanisms include stimulation of hematopoietic stem and progenitor cells, protection from oxidative and inflammatory damage within the marrow microenvironment, and normalization of cytokine networks controlling immune cell production and survival (Zheng et al. 2023). Although these findings are encouraging, most evidence is derived from preclinical models, and differences in formulations, dosing regimens, and study designs limit direct translation to clinical practice. Further well-designed clinical trials are required to confirm their efficacy in therapy-induced leukopenia and immunosuppression.

### Cancer- and chemotherapy- induced immunosuppression

In chemotherapy-associated immunosuppressive states, *Astragalus membranaceus* enhances innate and adaptive immune responses through activation of macrophages, dendritic cells, and lymphocytes, leading to improved immune recovery and reduced leukopenia in chemotherapy-treated patients (Cho and Leung 2007; Yang et al. 2013). Collectively, these immunomodulatory effects are reflected in clinical observations, where improved total WBC counts, enhanced lymphocytes and NK activity, and reduced severity of leukopenia have been reported in cancer patients receiving a chemotherapy adjunct in randomized/non-randomized cancer trials (Li et al. 2023b). Furthermore, *Ganoderma lucidum* β-glucans/triterpenes activate macrophages/DCs via dectin-1/TLR2/4 (enhancing phagocytosis/IL-12/TNF-α) and stimulate NK cytotoxicity/CD3^+^/CD4^+^ for antitumor surveillance (Matsui et al. 2001; Borchers et al. 2008) (Borchers et al. 2008; Matsui et al. 2001), increasing CD3^+^, CD4^+^ and NK cells, improving QOL scores, and reducing infection rates in chemo-/radiotherapy patients (Borchers et al. 2008; Gao and Homayoonfal 2023). *Panax ginseng* ginsenosides enhance NK activity, macrophage and DC maturation, and Th1/Th2 balance (increases IFN-γ/IL-2/T-cell responses) (Kim et al. 2022), reducing fatigue with modest NK increases and better immune parameters in cancer/post-chemo fatigue (Lee et al. 2025). Despite these promising results, the available clinical evidence remains heterogeneous, with considerable variation in study populations, treatment protocols, and outcome measures. In addition, many studies have relatively small sample sizes, making it difficult to draw definitive conclusions regarding their routine use as adjunctive therapies in oncology settings.

### Respiratory infections and mucosal immunity

In respiratory infections, *Echinacea* polysaccharides and alkamides stimulate macrophage and neutrophil phagocytic activity and modulate IL-1β, TNF-α and IL-10 production via CB2 and NF-κB signaling, while also enhancing NK cell activity and mucosal IgA (Hudson and Vimalanathan 2011; Dapas et al. 2014), these combined immunomodulatory effects have been associated with reduced incidence and duration of URI although randomized controlled trial (RCT) findings remain heterogeneous with respect to mucosal immune markers (Hudson and Vimalanathan 2011; Dapas et al. 2014). Similarly, *Sambucus nigra* (elderberry) anthocyanins inhibit viral entry while increases IL-6, IL-8 and TNF-α production from monocytes with antioxidant and NK-supporting effects (Roschek et al. 2009; Hawkins et al. 2019) (Roschek et al. 2009; Tiralongo et al. 2016; Hawkins et al. 2019), clinically these actions correlate with shortened influenza-like illness duration and improved symptom severity scores (Hawkins et al. 2019) (Tiralongo et al. 2016; Hawkins et al. 2019). In a complementary manner, *Andrographis paniculata* andrographolide decreases excessive NF-κB cytokines while enhancing phagocytic activity, IL-12 expression and Th1/T-cell responses (Aromdee et al. 2011; Islam et al. 2018; Zeng et al. 2022). Accordingly, it has been shown to improve acute respiratory infection symptoms and inflammatory markers with a generally favorable safety profile (Islam et al. 2018; Zeng et al. 2022).

### Autoimmune and chronic inflammatory disorders

Curcumin, resveratrol, and EGCG exert broad immunoregulatory effects through modulation of inflammatory signaling pathways and restoration of immune homeostasis, contributing to improvements in inflammatory biomarkers and disease activity across several autoimmune and chronic inflammatory disorders. Clinical studies have reported beneficial effects in conditions such as rheumatoid arthritis (RA), inflammatory bowel disease (IBD), and psoriasis, although the available evidence remains limited by relatively small sample sizes and study heterogeneity (Aggarwal and Harikumar 2009; Gupta et al. 2011; Minagawa et al. 2015; Ghoushi et al. 2024).

### General immune enhancement

In the context of general immune enhancement, β-glucans from medicinal mushrooms such as maitake, shiitake, and reishi engage pattern-recognition receptors including dectin-1, CR3, and Toll-like receptors, thereby promoting phagocytosis, ROS generation, and cytokine production, while also enhancing NK cell activity and mucosal IgA responses (Matsui et al. 2001; Borchers et al. 2008), These immunostimulatory effects are reflected in increased NK cell activity, improved CD4^+^/CD8^+^ ratios, and elevated salivary IgA levels in both healthy and mildly immunocompromised individuals. Likewise, propolis-derived flavonoids enhance phagocytic activity and modulate pro-inflammatory cytokines, including TNF-α, IL-1β, and IL-6, while providing additional mucosal antioxidant protection. These effects are associated with reduced incidence and severity of respiratory and oral infections (Sforcin 2016). Multi-herb formulas synergistically modulate neutrophils, NK cell and T-cell activity as well as cytokines, leading to increased leukocyte counts and improved vaccine responses despite formulation variability (Zheng et al. 2023; Pillai and Antony 2024; Kim et al. 2025).

### Herbal adjuvants for vaccine response enhancement in immunocompromised hosts

Herbal immunomodulators show emerging promise as vaccine adjuvants, particularly in immunocompromised populations where conventional vaccine responses are suboptimal (Alanazi et al. 2023). Supporting this, A 2024 RCT provided preliminary evidence that a *Codonopsis pilosula* and *Angelica sinensis* herbal complex may improve T-lymphocyte counts, accelerate WBC recovery, and overall improve immune function in NSCLC patients receiving chemotherapy and radiotherapy (Cho et al. 2024). Notably, the intervention enhanced post-treatment immune reconstitution, suggesting potential as a vaccine adjuvant in oncology settings where immunosuppression limits seroconversion (Cho et al. 2024).

In parallel, the COVER study (NCT05080218), an ongoing prospective trial, specifically evaluates herbal immunomodulators in rheumatic disease patients receiving COVID-19 boosters, measuring antibody titers and cellular immunity despite immunosuppressive therapy (ClinicalTrials.gov., 2024). Although still preliminary, unpublished data from the ongoing COVER study (NCT05080218) suggest improved humoral responses compared to controls, highlighting the potential of herbal intervention to overcome vaccine hyporesponsiveness in autoimmune conditions (ClinicalTrials.gov., 2024). These findings position herbal immunomodulators as valuable adjuncts to vaccination strategies in transplant recipients, elderly populations, and patients with chronic diseases. While these findings are encouraging, current evidence remains preliminary, and the reliance on limited clinical studies and ongoing trials highlights the need for larger, well-controlled investigations before definitive recommendations can be made.

### Long COVID and post-viral immune dysregulation

Long COVID represents a novel indication for herbal immunomodulators targeting persistent immune dysregulation, microbial dysbiosis, and chronic inflammation following SARS-CoV-2 infection (Alanazi et al. 2023). A 2023 systematic review identified *Astragalus membranaceus*, *Andrographis paniculata*, and *Glycyrrhiza uralensis* as particularly effective in reversing post-viral immune exhaustion through multiple mechanisms. For instance, APS restored CD4^+^ T-cell function and normalized cytokine profiles in COVID-19 recovery cohorts, whereas *Andrographis* reduced persistent low-grade inflammation and fatigue scores (Akter et al. 2023).

Furthermore, small clinical case series published in 2024 reported early clinical observations suggesting that multi-herb protocols may improve Long COVID symptoms, with objective improvements in NK cell activity and gut microbiome diversity (Alanazi et al. 2023). These herbs address the “post-viral immunometabolic syndrome” by simultaneously supporting exhausted adaptive immunity, modulating persistent innate activation, and restoring microbial homeostasis disrupted by both infection and antiviral therapies (Alanazi et al. 2023). At present, evidence in Long COVID remains limited, with most available data derived from small observational studies and indirect evidence from related post-viral syndromes. Therefore, larger randomized clinical trials are required to establish efficacy, optimal dosing strategies, and long-term safety.

### Sepsis and acute cytokine storm syndromes

Sepsis and cytokine release syndromes represent acute, life-threatening conditions where herbal immunomodulators offer rapid bidirectional immune modulation (Nisar et al. 2023). A 2023 meta-analysis of early-phase clinical trials reported that quercetin, resveratrol, and curcumin were associated with reductions in serum TNF-α, IL-6, and IL-1β levels in sepsis patients while preserving essential antimicrobial responses. Clinically, intravenous quercetin formulations shortened mechanical ventilation duration and reduced mortality as compared to standard care (Nisar et al. 2023). Similarly, *Andrographis paniculata* extracts, standardized to andrographolide, showed comparable efficacy to low-dose corticosteroids in early sepsis, rapidly downregulating NF-κB-driven cytokine storms while maintaining monocyte phagocytic capacity (Prabhakornritta et al. 2025).

### Autoimmune diabetes and vaccine-associated type 1 diabetes mellitus (T1DM)

The intersection of vaccination, autoimmunity, and metabolic disorders represents an emerging area of interest for herbal immunomodulators, particularly following reports of post-COVID-19 vaccination autoimmune diabetes. A 2023 systematic review analyzed 47 cases of new-onset T1DM temporally associated with COVID-19 vaccination, identifying dysregulated Th1/Th17 responses and β-cell autoantibody surges as key mechanisms. The authors proposed herbal immunomodulators as preventive/therapeutic adjuncts due to their established ability to restore Treg/Th17 balance and suppress pathogenic autoantibody production without broad immunosuppression (Alsudais et al. 2023).

Among these agents, curcumin has shown particular promise based on mechanistic overlap with pathways implicated in post-vaccine autoimmunity. Preclinical studies suggest that curcumin may support immune regulation in autoimmune diabetes through modulation of inflammatory pathways and restoration of immune tolerance, leading to reduced insulitis and preservation of β-cell function (Joshi et al. 2022). Emerging clinical evidence suggests that curcumin formulations, including nano-curcumin, may exert immunomodulatory effects in early type 1 diabetes; however, these observations remain preliminary and require confirmation in larger, well-controlled clinical trials (Joshi et al. 2022).

In addition, APS have demonstrated hematopoietic, anti-apoptotic, and immunomodulatory effects relevant to diabetes-associated immune dysfunction. In STZ-induced diabetic models, APS administration was shown to restore splenic Treg cell populations, reduce pancreatic inflammatory infiltration, and improve fasting glucose levels, effects that were mechanistically linked to modulation of the TLR4/NF-κB signaling pathway (Guo et al. 2023). While these findings provide important mechanistic insights, clinical evidence supporting APS effects on β-cell stress in at-risk human populations remains limited and largely preliminary.

### Modulation of glycemic control and insulin sensitivity in type 2 diabetes mellitus (T2DM)

**Fig. 3:**
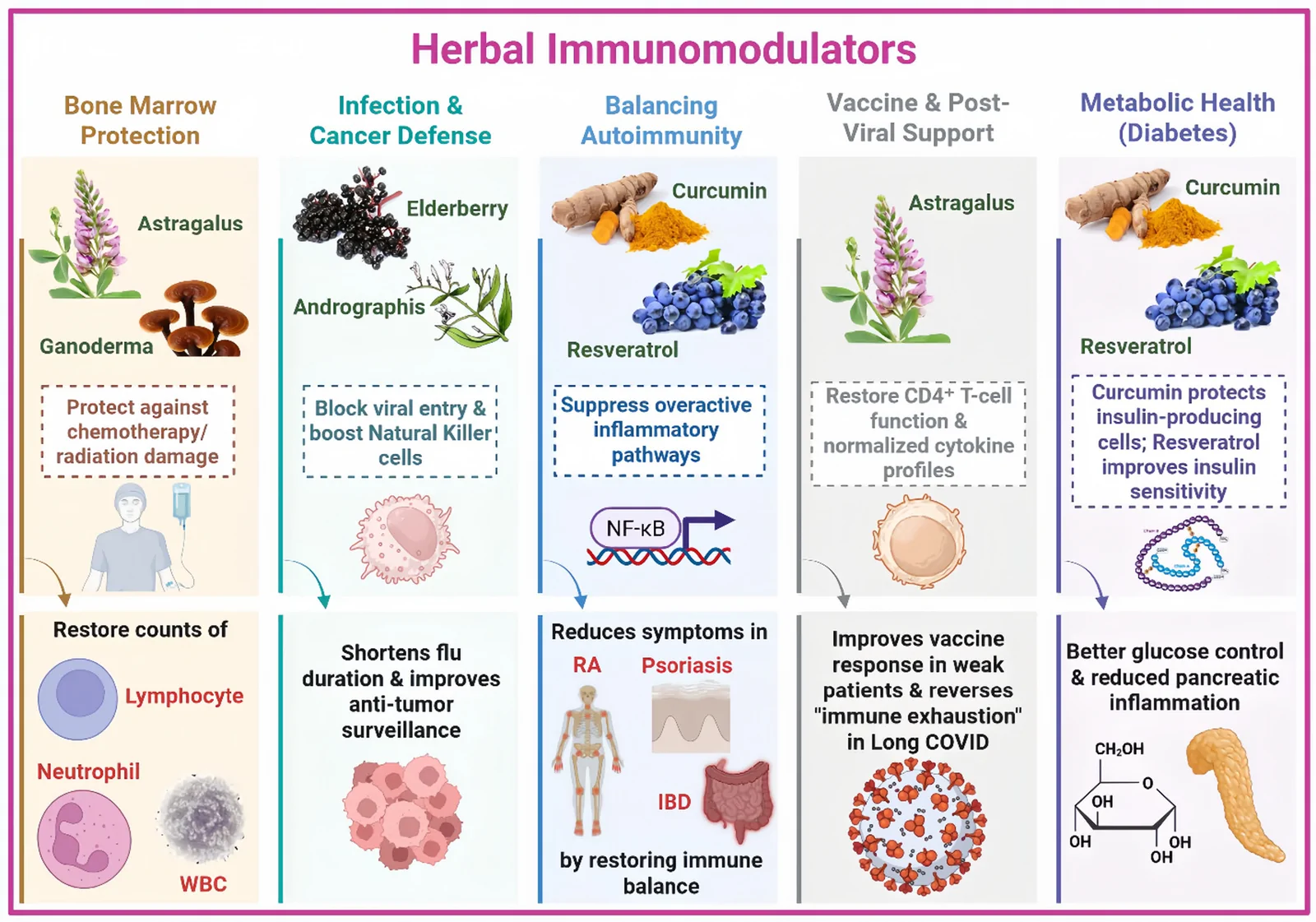
Therapeutic applications of immunomodulatory phytocompounds in the restoration and regulation of immune function across diverse disease states. The figure summarizes the potential roles of selected phytocompounds in supporting immune recovery, maintaining immune homeostasis, and improving disease outcomes in immune-related disorders. Created using BioRender.com

Resveratrol has demonstrated clinically relevant effects on glycemic regulation and insulin sensitivity in patients with type 2 diabetes. A systematic review and meta-analysis of nine RCTs (283 T2DM patients) demonstrated that resveratrol supplementation reduced fasting plasma glucose by − 0.29 mmol/L (95% CI − 0.51, − 0.06; *p* < 0.01) and insulin resistance (HOMA-IR) by − 0.52 (95% CI − 0.92, − 0.12; *p* = 0.01) compared to placebo (Zhu et al. 2017). Subgroup analysis revealed dose-dependent effects, with resveratrol ≥ 100 mg/day producing superior fasting glucose reductions (− 0.46 mmol/L) versus lower doses (Zhu et al. 2017). No significant effects on HbA1c, fasting insulin, or lipid profiles were observed, suggesting resveratrol’s primary benefit targets acute glycemic regulation and insulin signaling rather than long-term glycemic memory (Zhu et al. 2017). Collectively, these findings suggest that resveratrol may serve as a promising adjunct for improving glycemic control and insulin sensitivity in T2DM. However, the relatively small number of participants and variability in study design warrant further large-scale clinical trials to confirm its long-term efficacy and safety (Fig. 3).

## Pharmacokinetics and bioavailability

The pharmacokinetics and bioavailability of phytoimmunomodulatory compounds critically influence their in vivo efficacy. Despite strong immunomodulatory activity in preclinical studies, many phytochemicals exhibit poor oral bioavailability due to low aqueous solubility, limited intestinal absorption, rapid metabolism, and extensive first-pass hepatic and intestinal biotransformation.

Polyphenols such as curcumin, resveratrol, and quercetin are particularly affected by these limitations. Curcumin shows extremely low systemic availability due to poor absorption and rapid conversion into glucuronide and sulfate metabolites, while resveratrol undergoes extensive phase II metabolism resulting in low circulating parent compound levels. Quercetin also exhibits variable and generally low bioavailability depending on its glycosylation form and intestinal transport mechanisms (Chelimela et al. 2024; Jihwaprani et al. 2024).

Moreover, polysaccharide-based immunomodulators such as Astragalus membranaceus polysaccharides are poorly absorbed due to their high molecular weight. However, they may exert systemic immunomodulatory effects indirectly via gut microbiota modulation and interaction with gut-associated lymphoid tissue (GALT) (Ye et al. 2023). Furthermore, terpenoids such as ginsenosides (*Panax ginseng*) and alkaloids such as berberine also display limited bioavailability due to poor intestinal permeability and active efflux via P-glycoprotein, as well as extensive first-pass metabolism, which significantly reduces their systemic concentrations (Thomas et al. 2021; Hu et al. 2023a). However, the bioavailability of phytoimmunomodulators can be enhanced through structural modification, metabolic pathway inhibition, P-gp modulation, gut microbiota regulation, and advanced delivery systems such as nanoparticles, liposomes, and phospholipid complexes (Thomas et al. 2021; Hu et al. 2023a).

## Safety, toxicity, and drug–herb interaction considerations

Although phytocompounds from medicinal plants have shown promising immunomodulatory effects, their clinical application must be carefully evaluated in terms of safety and potential risks. Phytocompounds often exert pleiotropic biological activities, but these effects may also lead to toxicity depending on factors such as dose, duration of use, extract composition, and formulation. Some phytocompounds have been reported to cause adverse effects, including hepatotoxicity, nephrotoxicity, gastrointestinal disturbances, or hematological toxicity when administered at high doses or in vulnerable populations (Başaran et al. 2022; Mugale et al. 2024).

In addition, drug–herb interactions remain a significant concern, especially in elderly patients and individuals who are concurrently receiving pharmacological treatments. Many phytocompounds can interact with conventional drugs through their effects on cytochrome P450 enzymes (CYP450) or P-glycoprotein, potentially altering drug metabolism and absorption. Such interactions may elevate or diminish the efficacy of immunosuppressive drugs, corticosteroids, anticoagulants, chemotherapeutic agents, or other therapies (Gómez-Garduño et al. 2022; Ajaghaku and Chinwuba 2025). Notably, quercetin exerted a dose-dependent inhibitory effect on CYP3A4 enzyme and drug transporters in preclinical studies, resulting in substantial pharmacokinetic modulation of cyclosporine. Dual behavior of some natural bioactive compounds (e.g., flavonoids have antioxidant and pro-oxidant activity) must be taken into consideration, being influenced by dosage, the presence of reduced metal ions (such as iron), and environmental factors (Ashrafpour and Ashrafpour 2025). Therefore, careful monitoring and evaluation of potential herb–drug interactions are crucial for ensuring patient safety (Gupta et al. 2025).

These safety concerns represent an important barrier to clinical translation, as variability in phytochemical composition, differences in manufacturing processes, and incomplete characterization of herb–drug interactions may compromise both efficacy and safety across patient populations. To ensure safe therapeutic use, future clinical studies should focus on standardizing phytocompound formulations, conducting thorough toxicity profiling, and performing drug–herb interaction studies (Rashid et al. 2025). Additionally, clinical trials should include diverse populations and evaluate the long-term effects and potential side effects of phytocompounds when used alongside conventional therapies (Nisar et al. 2023).

## Limitations and future directions

Despite increasing evidence supporting the immunomodulatory potential of phytocompounds, their clinical translation remains constrained by several limitations (Van Norman 2020; Ali et al. 2021). While promising preclinical data from in vitro and animal studies suggest significant therapeutic effects, the predominance of preclinical findings means that these results may not accurately predict human immune responses (Van Norman 2020; Karnik et al. 2023). Because in vitro and animal models often fall short of accurately simulating the intricacies of the immune system of humans, particularly in the setting of chronic illnesses, this gap between preclinical and clinical effectiveness is a major barrier. It is so often difficult to apply these results to therapeutic practice. Clinical information about the safety and efficacy of phytocompounds in humans is currently lacking. Research often uses small sample sizes, which reduces the statistical power and generalizability of the findings (Karthikeyan et al. 2025). Additionally, short follow-up durations complicate the understanding of long-term effects and potential adverse events that might arise from prolonged use (Cuzick 2023). Moreover, the heterogeneity in study design across clinical trials, including differences in outcome measures, dosing regimens, and patient populations, makes it difficult to compare and draw conclusions across studies (Lipkovich et al. 2024). This variability in methodological approaches leads to inconsistent results and limits the ability to form generalizable clinical recommendations. In addition to scientific and clinical limitations, the incorporation of phytocompounds into routine clinical practice remains hindered by several translational challenges. Many phytochemicals exhibit poor bioavailability, limited stability, and variable pharmacokinetic profiles, which may restrict their therapeutic efficacy in humans despite promising preclinical findings. Furthermore, the lack of standardized extraction methods, phytochemical characterization, and formulation protocols contributes to substantial variability among studies and commercial products. Potential herb–drug interactions, particularly those involving cytochrome P450 enzymes and drug transporters, represent an additional safety concern, especially in patients receiving multiple medications. Regulatory challenges, including quality control requirements and the absence of harmonized approval frameworks, further complicate the clinical development and acceptance of phytocompound-based therapies (Chihomvu et al. 2024; Mullick et al. 2026).

To address these issues, future clinical research must prioritize large-scale randomized controlled trials (RCTs) that incorporate diverse patient populations (He et al. 2020b). Larger sample sizes will enhance the statistical power of studies, allowing for more accurate assessments of safety and efficacy (He et al. 2020b). Additionally, standardized protocols for study design, including uniform dosing regimens, outcome measures, and patient inclusion criteria, should be established to improve the consistency and reliability of clinical trials (He et al. 2020b).

Furthermore, long-term follow-up studies are essential for capturing potential adverse effects and toxicities associated with the prolonged use of phytocompounds. Studies should also include comprehensive toxicity profiling to assess the full spectrum of potential side effects, including hepatic, renal, and gastrointestinal toxicity that may not be apparent in shorter trials (Pham et al. 2020). Another critical aspect that has been insufficiently addressed is the comparative effectiveness of phytocompounds versus conventional immunotherapies. Comparative studies are necessary to determine whether phytocompounds offer additional benefits over current standard treatments or pose risks when used in conjunction with other therapies (Pham et al. 2020).

Multi-omics approaches (e.g., genomics, proteomics, metabolomics) represent an emerging strategy to gain deeper insights into the mechanisms of action of phytocompounds. These approaches can provide a more holistic understanding of how phytocompounds interact with the immune system at a molecular level, and they hold the potential to identify biomarkers for personalized medicine (Pham et al. 2020; Zhao et al. 2024a).

Moreover, although numerous phytocompounds have demonstrated promising immunomodulatory effects in experimental models, robust clinical validation remains limited. Additional large-scale, multicenter clinical trials are required to establish efficacy, safety, optimal dosing regimens, and long-term outcomes across different patient populations.

Moving forward, to facilitate the regulatory approval and clinical translation of phytocompounds, it is crucial to standardize their preparations, ensuring consistent quality, potency, and purity across formulations. Rigorous pharmacokinetic and toxicity evaluations must be conducted to support the safe use of these compounds and optimize patient outcomes in clinical settings (Karthikeyan et al. 2025).

## Conclusion

Immune homeostasis is essential for host defense, immune surveillance, and physiological balance, and its disruption contributes to a wide spectrum of immune-related disorders. Despite significant advances in immunotherapy, current treatment strategies remain limited by adverse effects, incomplete efficacy, resistance, and high cost, underscoring the need for safer and more effective immunomodulatory approaches. This review provides an integrative overview of immune disorder pathophysiology, associated risk factors, and current therapeutic strategies, with a particular focus on the immunomodulatory potential of plant-derived bioactive compounds. Phytocompounds including polysaccharides, flavonoids, polyphenols, terpenoids, and alkaloids exert pleiotropic effects on both innate and adaptive immunity through modulation of key immune cells and signaling pathways, such as NF-κB, MAPKs, JAK/STAT, and Nrf2. Emerging preclinical and clinical evidence supports their potential role in managing autoimmune, inflammatory, infectious, and immune-mediated metabolic disorders. Collectively, these findings highlight phytocompounds as promising candidates for immune modulation, offering a multifaceted and potentially safer alternative to conventional therapies. However, their successful clinical translation requires addressing critical challenges related to bioavailability, standardization, and variability in clinical evidence. Future research should prioritize well-designed large-scale clinical trials, advanced delivery systems, and mechanistic studies to fully realize their therapeutic potential.

## Data Availability

All data are available within the manuscript.

## Abbreviations

4T1: Murine breast cancer cell line
5-FU: 5-Fluorouracil
Ag-Ab: Antigen-antibody
AhR: Aryl hydrocarbon receptor
AIM2: Absent in melanoma 2
AKI: Acute kidney injury
Akt: Protein kinase B
ALI: Acute lung injury
ALT: Alanine aminotransferase
anti-PD-L1: Anti-programmed death-ligand 1 antibody
anti-SRBC: Anti-sheep red blood cell antibodies
AOM/DSS: Azoxymethane/dextran sulfate sodium
AP-1: Activator protein-1
APS: Astragalus polysaccharides
Arg-1: Arginase-1
AST: Aspartate aminotransferase
BALF: Bronchoalveolar lavage fluid
Bcl-2: B-cell lymphoma-2
Beclin-1: Autophagy-related protein
BMDMs: Bone marrow-derived macrophages
BMECs: Brain microvascular endothelial cells
BTK: Bruton tyrosine kinase
C3G: Cyanidin-3-glucoside
CACNB2: Calcium voltage-gated channel subunit beta 2
CALB: Candida antarctica Lipase B
CAR T cell: Chimeric antigen receptor T-cell
CARD9: Caspase recruitment domain-containing protein 9
CAT: Catalase
CCL2: C-C motif chemokine ligand 2
CCR1: Chemokine receptor 1
CD: Cluster of differentiation
CD244/PD-1: Cluster of differentiation 244/programmed death-1
CD274/PD-L1: Cluster of differentiation 274/programmed death-ligand 1
CD38 + NK: CD38-positive natural killer cells
CD40: Cluster of differentiation 40
CD40L: CD40 ligand
CGD: Chronic granulomatous disease
cGVHD: Chronic graft-versus-host disease
CIA: Collagen-Induced arthritis
Clec4e/Mincle: C-type lectin receptor
cNOS: Constitutive nitric oxide synthase
CNS: Central nervous system
COVID: Coronavirus disease
COX-2: Cyclooxygenase-2
CR3: Complement receptor 3
CRISPR-Cas9: Clustered regularly interspaced short palindromic repeats-associated protein 9
cSCC: Cutaneous squamous cell carcinoma
CTL: Cytotoxic T lymphocyte
CTLA-4: Cytotoxic T-lymphocyte-associated protein 4
CUMS: Chronic unpredictable mild stress
CXCL10: C-X-C motif chemokine ligand 10
CY: Cyclophosphamide
DCs: Dendritic cells
DLL4: Delta-like ligand 4
DRP1: Dynamin-related protein 1
DTH: Delayed-type hypersensitivity
DZN: Diazinon
E. coli: Escherichia coli
EAE: Experimental autoimmune encephalomyelitis
EBV: Epstein–barr virus
ECAR: Extracellular acidification rate
ECRS: Eosinophilic chronic rhinosinusitis
EGCG: Epigallocatechin gallate
EGFR: Epidermal growth factor receptor
ER: Endoplasmic reticulum
ERK: Extracellular signal-regulated kinase
FBG: Fasting blood glucose
Fizz1: Found in inflammatory zone-1
fMLF: N-formylmethionyl-leucyl-phenylalanine
FMT: Fecal microbiota transplantation
FN14: Fibroblast growth factor-inducible 14
FOXP3: Forkhead box P3
FPR1: Formyl peptide receptor 1
GATA3: GATA binding protein-3
GPX-1: Glutathione peroxidase-1
GR: Glutathione reductase
GSH-Px: Glutathione peroxidase
H1N1: Influenza A virus H1N1
H9N2: Influenza A virus
HaCaT: Human keratinocyte cell line
Hes1: Hairy and enhancer of split-1
HIF-1α: Hypoxia-inducible factor-1 alpha
HIV: Human immunodeficiency virus
HMGB1: High mobility group box 1
Hmox: Heme oxygenase
HO: Heterotopic ossification
HO-1: Heme oxygenase-1
HOEPC: Human outgrowth endothelial progenitor cells
HSCT: Hematopoietic stem cell transplantation
HSE: Herpes simplex encephalitis
IBD: Inflammatory bowel disease
ICAM-1: Intercellular adhesion molecule-1
IDDM: Insulin dependent diabetes mellitus
IDO1: Indoleamine 2,3-dioxygenase 1
IFN-γ: Interferon gamma
Ig: Immunoglobulin
IGFBP-1: Insulin-like growth factor-binding protein-1
IgG2a: Immunoglobulin G2a
IKKβ: Inhibitor of nuclear factor kappa-B kinase beta
IL: Interleukin
IL1R1: Interleukin-1 receptor type 1
IMQ: Imiquimod
iNOS: Inducible nitric oxide synthase
IR: Ischemia-reperfusion
IRF: Interferon regulatory factor
ITP: Immune thrombocytopenic purpura
iTreg: Induced regulatory T cells
IVIG: Intravenous immunoglobulin
IκBα: Inhibitor of kappa B alpha
JAK: Janus kinase
JNK: c-Jun N-terminal kinase
Keap1: Kelch-like ECH-associated protein 1
Kyn: Kynurenine
LAD: leukocyte adhesion deficiency
LC3: Microtubule-associated protein 1 light chain 3
LC3A/B: Microtubule-associated protein 1 light chain 3 A/B
LC3II/LC3I: Microtubule-associated protein 1 light chain 3 II/I
LCK/AKT: Lymphocyte-specific protein tyrosine kinase/protein kinase B
LCs: Langerhans cells
LPS: Lipopolysaccharide
LSD1: Lysine-specific demethylase-1
LTB4: Leukotriene B4
LUAD: Lung Adenocarcinoma
mAbs: Monoclonal antibodies
MAPK: Mitogen-activated protein kinase
MCAO: Middle cerebral artery occlusion
MCP-1: Monocyte chemoattractant protein-1
MDA: Malondialdehyde
MDCK: Madin-Darby canine kidney cells
MHC: Major histocompatibility complex
MMP: Matrix metalloproteinase
MNCs: Mononuclear cells
MPA: Mycophenolic acid
MPO: Myeloperoxidase
mRNA: Messenger ribonucleic acid
MS: Multiple sclerosis
Mtb: Mycobacterium tuberculosis
mTOR: Mechanistic target of rapamycin
MTX: Methotrexate
Mx1: Myxovirus resistance protein 1
MyD88: Myeloid
NADPH: Nicotinamide adenine dinucleotide phosphate
NAMPT: Nicotinamide phosphoribosyl transferase
NEMO: NF-kB essential modulator
NFAT: Nuclear Factor of Activated T cells
NF-κB: Nuclear factor kappa-B
NK: Natural killer
NKG2D: Natural killer group 2, member D
NKp30: Natural Killer protein 30
NLRP3: Nod-like receptor protein 3
NO: Nitric oxide
NOD: Non-obese diabetic
NOD-2: Nucleotide-binding oligomerization domain-containing protein 2
Nos2: Nitric oxide synthase-2
NQO1: NAD(P)H quinone oxidoreductase 1
Nrf2: Nuclear factor erythroid 2-related factor 2
NSCLC: Non-small cell lung cancer
OA: Osteoarthritis
OPG: Osteoprotegerin
OSN: Olfactory sensory neurons
P. aeruginosa: Pseudomonas aeruginosa
PAD: Peptidyl-arginine deiminase
PBMCs: Peripheral blood mononuclear cells
PD-L1: Programmed death-ligand 1
PFC: Prefrontal cortex
PFKL: Phosphofructokinase, liver type
PGE_2_: Prostaglandin E_2_
PI3K: Phosphatidylinositol 3-kinase
PKM2: Pyruvate kinase M2
PKR: Protein kinase R
PMVECs: Pulmonary microvascular endothelial cells
PNS: Peripheral nervous system
PPARγ: Peroxisome proliferator-activated receptor gamma
PRL: Prolactin
PTEN: Phosphatase and tensin homolog
QOL: Quality of life
RA: Rheumatoid arthritis
RANK: Receptor activator of nuclear factor kappa
RANKL: Receptor activator of nuclear factor kappa-B ligand
RASF: Rheumatoid arthritis synovial fibroblasts
RCT: Randomized controlled trial
RGAP: Red ginseng acidic polysaccharide
RIG-I: Retinoic acid-inducible gene I
RORγT: Retinoic acid receptor-related orphan receptor-gamma t
ROS: Reactive oxygen species
S. aureus: Staphylococcus aureus
S1PR: Sphingosine 1-phosphate receptor
SARS-CoV-2: Severe acute respiratory syndrome coronavirus 2
SCFA: Short-chain fatty acid
SCID: Severe combined immunodeficiency
SIC: Sepsis-induced coagulopathy
SIRS: Systemic inflammatory response syndrome
SIRT1: Sirtuin 1
SLE: Systemic lupus erythematosus
SOCS: Suppressors of cytokine signaling
SOD: Superoxide dismutase
SP-D: Surfactant protein D
SS: Sjögren’s syndrome
STAT: Signal transducer and activator of transcription
STING: Stimulator of interferon genes
STZ: Streptozotocin
Syk: Spleen tyrosine kinase
T1DM: Type 1 diabetes mellitus
TAMs: Tumor-associated macrophages
T-AOC: Total antioxidant capacity
TB: Tuberculosis
T-bet: T-box expressed in T cells
TD: T cell-dependent
TGF-β: Transforming growth factor-β
Th: T helper
TI: T cell-independent
Ti: Titanium
TIMP: Tissue inhibitor of metalloproteinases
TLRs: Toll-like receptors
TNBC: Triple-negative breast cancer
TNF: Tumor necrosis factor
TPO-RAs: Thrombopoietin receptor agonists
TRADD: TNF receptor-associated death domain
TRAF3/6: TNF receptor-associated factor 3 / TNF receptor-associated factor 6
Treg: Regulatory T cell
TRIF: TIR domain-containing adaptor inducing IFN-β
Trp: Tryptophan
TSLP: Thymic stromal lymphopoietin
TUNEL: Terminal deoxynucleotidyl transferase dUTP nick end labeling
TWEAK: Tumour necrosis factor-like weak inducer of apoptosis
TXB2: Thromboxane B2
URI: Upper respiratory infection
URSA: Unexplained recurrent spontaneous abortion
VCAM-1: Vascular cell adhesion molecule-1
WAS: Wiskott-Aldrich syndrome
WBC: White blood cell
ZO-1: Zonula Occludens-1
